# Winter Temperature Affects Fatty Acid Composition and Gene Expression, but Not Fat Content and Survival in a Northern Population of a Range‐Expanding Spider

**DOI:** 10.1002/ece3.72507

**Published:** 2025-11-27

**Authors:** Carolina Ortiz‐Movliav, Marina Wolz, Michael Klockmann, Andreas Walter Kuss, Lars Riff Jensen, Corinna Jensen, Alexander Wacker, Gabriele B. Uhl

**Affiliations:** ^1^ General and Systematic Zoology, Zoological Institute and Museum University of Greifswald Greifswald Germany; ^2^ Bavarian Environment Agency Biodiversity Centre Rhön Bischofsheim i.d. Rhön Germany; ^3^ Center for Functional Genomics of Microbes University of Greifswald Greifswald Germany; ^4^ Interfaculty Institute for Genetics and Functional Genomics University of Greifswald Greifswald Germany; ^5^ Animal Ecology, Zoological Institute and Museum University of Greifswald Greifswald Germany

**Keywords:** climate change, cold‐adaptation, fatty acids, gene expression, overwintering, range‐expansion

## Abstract

Species expand their geographic distribution when environmental conditions are favorable or when mutations arise that allow them to live in previously unfavorable conditions. The European wasp spider, *Argiope bruennichi* , has expanded its range poleward, and populations at the northern edge show higher tolerance to cold and are genetically differentiated from the core populations. We aimed to investigate the degree and limits of plasticity in a recently cold‐adapted Estonian population by exposing overwintering juveniles (spiderlings) to three fixed winter regimes over the course of three months. These regimes differed in absolute and relative day and night temperature: cold (5°C day, −15°C night), moderate (5°C day, −5°C night), and warm (15°C day, −5°C night). We expected a differential response to the winter regimes in survival, lipid content, metabolites, and gene expression patterns. The survival probability of the spiderlings decreased over winter by approximately 20% and their lipid content by 28%, with no significant differences between groups. Spiderlings also did not differ in content of saturated and monounsaturated fatty acids per dry weight. However, in spiderlings exposed to the warm winter regime, short‐chain omega‐3 PUFAs were less abundant (~57%) and long‐chain omega‐3 PUFAs more abundant (~66%) compared to the other regimes. The gene expression response was low under the cold regime and much higher under the warm regime, as compared to the moderate regime. The affected pathways suggest a more pronounced stress response under warmer winter temperatures. Taken together, our findings demonstrate that *A. bruennichi* spiderlings from a northern population can endure very different winter regimes. However, the observed physiological responses to the warmer regime suggest metabolic costs that may reduce spiderling survival probability after emergence from the egg sac. We conclude that, despite remarkable tolerance to different winter regimes, warmer winters have nuanced effects on spiderling physiology beyond survival probability.

## Graphical abstract

This study provides insight into the performance of a range‐expanding species and the physiological and molecular mechanisms underlying thermal tolerance at its expanding edge.

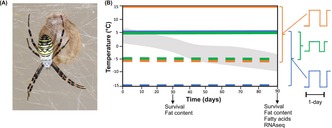

## 1 Introduction

With global climate change, temperatures have risen especially in the high‐latitude regions, and will continue to do so under all climate change projections (Ono et al. 2022). Winter temperatures in these regions are also rising faster than in any other season (Hahn et al. 2023), leading to reduced snow cover, potentially exposing overwintering arthropods to more extreme temperature fluctuations, since snow normally insulates and buffers against rapid temperature changes (Bale and Hayward 2010). The increasing frequency of extreme temperature events (Renault et al. 2022) also poses a significant threat to cold‐adapted species (Shirey et al. 2024). Understanding how these temperature extremes affect the physiology of overwintering organisms will help assess and predict their resilience in a changing climate.

Spiders, like other ectotherms, are directly affected by ambient temperature fluctuations (Harvey and Dong 2023), which influence their thermal performance and vulnerability to climate change (Burraco et al. 2020; Renault et al. 2022). However, as spiders have lower metabolic rates than expected from their body mass (Anderson 1970; Schmitz 2016), responses to temperature in spiders may differ from those of insects. Although extensive research exists on insect thermal physiology (e.g., Bale and Hayward 2010; Paaijmans et al. 2013; Harvey et al. 2020; Roberts et al. 2023), temperature effects in spiders remain understudied. Therefore, research on the susceptibility to temperature regimes is crucial to understanding spider thermal physiology under climate change and to facilitate informed comparisons with insects. We aim to contribute to filling this knowledge gap by studying the responses of spiders to different temperature regimes during winter. Understanding spider winter physiology is essential for gaining insights into how spiders cope with the degree of resilience to seasonal conditions (Schaefer 1977).

**FIGURE 1:**
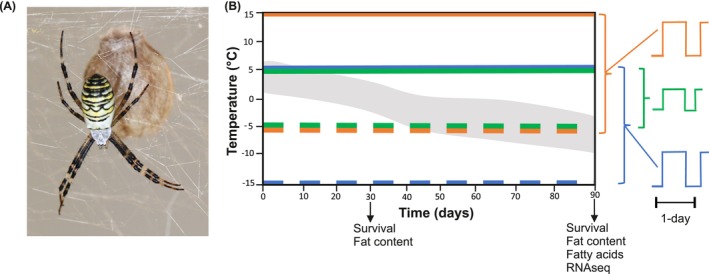
(A) *Argiope bruennichi* female with a silk‐woven egg sac (photo by G. Uhl) (B) Experimental design: Three temperature regimes were used that differed in mean temperatures and day‐night fluctuation. Solid horizontal lines represent day temperatures and short‐dashed horizontal lines represent night temperatures. Blue = cold regime (5°C day/−15°C; *n* = 49), green = moderate regime (5°C day/−5°C night, *n* = 52), orange = warm regime (15°C day/−5°C night; *n* = 52). The diurnal temperature amplitude of cold and warm regimes was higher compared to the moderate regime (illustration on the right of the graph). The shaded gray band represents a simulation of the average day and night temperatures in the shade over 3 months in Pärnu, Estonia [“natural” temperature data derived from climate robot (https://www.weatheronline.co.uk/) averaged over ten years (2004–2014)]. After one month of exposure, we measured survival and fat content and after three months we additionally explored the fatty acid profiles and differential gene expression (vertical arrows).

The European Wasp Spider, *Argiope bruennichi* (Scopoli, 1772) (Araneae: Araneidae) provides a valuable model for exploring how temperature affects performance and physiology. This species has undergone a remarkably rapid poleward range expansion from the Mediterranean to Scandinavia and into the Baltic region since the 1950s (Kumschick et al. 2011; Krehenwinkel et al. 2016; Wawer et al. 2017), a phenomenon commonly attributed to climate change (Hickling et al. 2006). However, the species' range expansion surpasses the rate of warming, reaching latitudes where global warming alone cannot fully explain its occurrence (Geiser 1997; Kumschick et al. 2011). The recently colonized northern habitats have much stronger seasonality, with shorter summers and colder winters than the Mediterranean core area. Interestingly, despite recent colonization, *A. bruennichi* from northern regions is already genetically differentiated from those of the core region (Krehenwinkel and Tautz 2013, Sheffer et al. in press), which might explain why adults are on average smaller, reproduce earlier, and early‐stage juveniles are less prone to disperse via ballooning (Wolz et al. 2020; Sheffer et al. in press). Further, spiderlings from the northern distribution prefer colder temperatures when given a choice (Krehenwinkel and Tautz 2013) and can cope with significantly lower temperatures during winter, determined by lower lethal temperatures and lower supercooling points (Sheffer et al. in press). Therefore, despite originating in the Mediterranean region, northern populations show signatures of cold adaptation and thus might be vulnerable to changing winter temperatures due to climate change. *A. bruennichi* overwinter as 1st instar juveniles (spiderlings) in an intricately woven egg sac placed in the vegetation (Figure 1A). They hatch from the egg already in autumn, molt once before winter and remain in the egg sac until spring. When the spiderlings emerge from the egg sac, they build orb webs for foraging and continue development by molting several times (Schaefer 1977). Although adult spiders can respond behaviorally to ambient temperatures, overwintering juveniles in the egg sac are exposed to the given winter conditions without the possibility of seeking more favorable microclimates.

The overwintering period often entails a dormant phase during which a species' vulnerability and physiological plasticity in response to temperature and daily fluctuation can be most accurately assessed, as has been done for insects (e.g., Williams, Hellmann, and Sinclair 2012). In temperate climates, daily temperature fluctuations during winter can severely compromise the metabolic and physiological activity of insects (Xu et al. 2017). High day temperatures (> 15°C) increase metabolic demands (Bale and Hayward 2010), while lower temperatures during the night (< 4°C) can trigger the use of lipids as well as carbohydrates and proteins to fuel the mechanisms that protect against cold (Sinclair 2015). The physiological processes active in insects during winter primarily focus on survival and preserving resources for growth in spring. This holds true especially for species with a fixed energy budget, i.e., that do not take in food during winter. Storage lipids (triacylglycerol fatty acids) thus serve as the primary energy source (Hahn and Denlinger 2011; Sinclair and Marshall 2018). Cold‐adapted overwintering insect species exhibit modifications in lipid composition, including a higher ratio of unsaturated to saturated fatty acids (Storey and Storey 2013), a pattern evident in the fatty acids incorporated into phospholipids of the cell membrane and in fatty acids used to store energy in the form of triacylglycerol and sterol esters (De Carvalho and Caramujo 2018). By increasing the proportion of mono‐ and polyunsaturated fatty acids, membrane fluidity can be maintained in colder environments (van Dooremalen and Ellers 2010; Sinclair and Marshall 2018). Regarding spiders, lipid loss in species that are active as juveniles during the winter has been investigated in a few studies (Ingle et al. 2018; Potts et al. 2020). Spiderlings that overwinter inside their egg sac with only the resources provided to the egg by the mother offer an opportunity to assess the physiological dynamics and adaptations at play, as well as the specific effects of varying winter regimes.

Our study aims to investigate the degree and limits of phenotypic plasticity of a recently cold‐adapted population of *A. bruennichi* at the northern edge of the distribution. We seek to explore whether the overwintering stage is resilient or susceptible to winter regimes that differ in absolute day and night temperature and in the degree of fluctuation in daily temperature. Specifically, we ask how winter temperature regimes affect survival and physiological responses in cold‐adapted *Argiope bruennichi* spiderlings, and whether a warmer winter regime imposes hidden metabolic costs with potential fitness consequences in light of ongoing climate change.

We subjected egg sacs containing spiderlings to one of three fixed winter regimes and measured how spiderling survival and physiology were affected in the course of winter. At two time points (after either 1 or 3 months of exposure) we opened the egg sacs and determined the survival proportion of the spiderlings and their total lipid content to gain insight into the dynamics of how spiderlings use energy stores under different winter regimes. Further, after 3 month exposure, we compared the spiderlings' fatty acid composition and their patterns of gene expression. Spiders may modulate the expression of genes in specific metabolic pathways under challenging winter conditions. By using comparative transcriptomics of gene‐regulated metabolites, we aimed to reveal metabolic differences between treatment groups that would otherwise require direct measurement (Hoedjes et al. 2024). We hypothesized that the winter regime would significantly affect the survival and physiology of spiderlings. Specifically, we predicted that high diurnal temperature fluctuations would be more challenging than moderate fluctuations, thus reducing survival probability. We hypothesized further that survivors would exhibit differential gene expression patterns depending on the experienced winter environment and predicted that the warmest treatment would result in the lowest survival probability and pronounced signatures of temperature stress in this cold‐adapted population.

## 2 Materials and Methods

### 2.1 Sampling and Winter Regimes

Adult mated female spiders of *Argiope bruennichi* (NCBI: txid94029) were collected in August 2017 from the edge of the distribution in Pärnu, Estonia (N: 58.294, E: 24.602) by GU. Three days after collection, the spiders were transferred to a climate‐controlled chamber at the University of Greifswald, Germany, and kept at 20°C, 70% relative humidity and a 14:10 h light: dark cycle. The females were housed in individual plastic containers (1000 mL) (*n* = 170), sprayed with water daily and fed regularly with blow flies (*Calliphora* sp.). When ovipositing, *A. bruennichi* females lay eggs in a silk‐woven egg sac (Figure 1A). After two oviposition events, the females were anesthetized with CO_2_ and preserved in ethanol. The egg sacs were moved to individual 5 × 5 × 3.5 cm plastic containers with mesh on opposite sides. Inside the egg sacs, the postembryos hatch from the eggs within two weeks after oviposition and molt once to first instar spiders (spiderlings) that remain in the egg sac over winter (Leborgne and Pasquet 2005). The spiderlings emerge from the egg sac in the spring of the following year and build individual webs.

On November 17th, 2017, a total of 153 egg sacs containing spiderlings were randomly assigned to three climate cabinets with fixed winter regimes. The egg sacs were sprayed with water each evening to mimic natural increases in nighttime humidity. The winter regimes differed in absolute day and night temperatures and the degree of diurnal temperature fluctuations: “cold” winter (5°C day/−15 C night; *n* = 49), “moderate” winter (5°C day/−5°C night; *n* = 52), and “warm” winter (15 C day/−5 C night; *n* = 52; Figure 1). The cold winter regime was maintained in a Percival LT‐36VL climate cabinet (CLF PlantClimatics GmbH, Wertingen, Germany), while the moderate and warm winter regimes were housed in Panasonic MLR‐352H climate cabinets (Ewald Innovationstechnik GmbH, Bad Nenndorf, Germany). Egg sacs were exposed to these three winter regimes for a duration of three months (Figure 1B). The cold regime featured low daytime temperatures just above freezing (5°C) and extreme nighttime cold (−15°C)—such diurnal fluctuations of 20 degrees do occasionally occur in Estonia. The moderate regime (5°C day/−5°C night) provided overall milder conditions, as e.g., occurs when there is snow cover. The warm regime represents a regime that does not occur in Estonia with warmer daytime temperatures (15°C) and colder nights (−5°C), more typical for the species southern populations, where occasional cold spells do occur. By subjecting the spiderlings to one of these regimes, we aimed to explore the resilience limit of this species and its physiological responses.

To assess how temperature affects the survival of the spiderlings during winter, we opened egg sacs at two time points, after one month (December 21, 2017, cold *n* = 21, moderate *n* = 24 and warm *n* = 34), and after three months of exposure to the winter regimes (February 19–23, 2018, cold *n* = 28, moderate *n* = 28 and warm *n* = 28). For each egg sac, we counted the number of live spiderlings, dead spiderlings, as well as unhatched/unfertilized eggs. We determined the survival proportion of the offspring as the number of live spiderlings divided by the total number of hatched spiderlings (live plus dead). The live spiderlings were flash frozen at −80°C and stored until further processing. Their fat content was evaluated for both exposure times (after 1 and 3 months), while fatty acids and differential gene expression were investigated after three months of exposure (Figure 1B).

### 2.2 Fat Content

From a subset of the total egg sacs (*n* = 125 out of 153), 30 spiderlings per egg sac were used for the analysis of the fat content (after a month: *n* = 17 cold, *n* = 18 moderate, *n* = 17 warm; after three months: *n* = 26 cold, *n* = 21 moderate, *n* = 26 warm). The fat content was obtained following the protocol described in Geiger et al. (2018). The samples were dried for 48 h at 60°C and then weighed (initial dry weight) using a high precision balance (Sartorius ME5, Sartorius AG, Göttingen, Germany). Subsequently, fat was extracted using 1 mL of acetone. After 48 h, the acetone was changed and left for another two days before removal. The samples were then dried again at 60°C for 48 h and subsequently weighed. The fat content was calculated as the weight difference between the two measurements. The relative fat content was calculated by dividing the fat content by the initial dry weight.

### 2.3 Fatty Acid Analysis

The extraction of fatty acids was performed on spiderlings from egg sacs that had experienced one of the three temperature regimes for three months. Eight egg sacs were randomly chosen from each regime, and a sample containing 15 spiderlings was collected from each egg sac. For fatty acid extraction, each sample was transferred to a glass vial containing 4 mL of dichloromethane‐methanol (2:1 v/v) and 50 μL nonadecanoic acid methyl ester (C19:0, 200 ng/μL) as an internal standard (Wacker et al. 2016). After storage for 48 h at −20°C, samples were placed in an ultrasonic bath for 7 s and the solvent was transferred to a new vial. A fresh dichloromethane‐methanol (2:1 v/v) solution was added to the sample and the closed vial was placed in the ultrasonic bath for 20 s. The solvent was collected and added to the solvent from the first extraction. The solvent was removed under a gentle stream of nitrogen gas by placing the vials in a thermostatically controlled concentrator evaporator at 40°C (Cole‐Parmer Stuart SBH130D/3) to prevent the reaction of lipids with atmospheric oxygen. The dried samples were resuspended in 4 mL 3 M/L of methanolic HCl (Sigma‐Aldrich Chemie, Darmstadt, Germany) and incubated for 20 min at 60°C in a closed vial to trans esterify fatty acids into fatty acid methyl esters (FAME). After cooling at 4°C for 20 min, the FAMEs were extracted three times. Each time, 1 mL of isohexane was added to the vials containing the samples in methanolic HCl and vortexed for 5 s before allowing the two phases to settle. This vortexing step was repeated three times. The isohexane phases of the three extractions were collected in a new vial. Subsequently, this fraction was evaporated to dryness under nitrogen at 40°C, resuspended in 100 μL of isohexane, transferred to GC vials with micro inserts, and stored at −20°C until injection. The FAMEs were analyzed by gas chromatography (6890 N, Agilent Technologies, Böblingen, Germany) according to Wacker and Weithoff (2009). Helium was used as carrier gas (1.5 mL/min) and FAMEs were separated on a DB‐225 column (Agilent, 30 m × 250 μm × 0.25 μm), applying an initial oven temperature of 60°C for 1 min, increasing at a rate of 20°C/min until 150°C, 10°C/min until 220°C and then held for 14 min. FAMEs were detected using a flame ionization detector (FID) at 250°C and quantified using multipoint standard calibration curves with HP chemstation (Agilent Technologies). To examine the identity of fatty acid methyl esters, mass spectra were recorded with a gas chromatograph (7890A, Agilent Technologies) connected to a mass spectrometer (Pegasus 4D GC‐TOFMS, LECO Instruments, Mönchengladbach, Germany). Data handling was carried out using associated chemstation software (6890 N, Agilent Technologies) and as described in Wacker et al. (2016). Fatty acids were identified on the basis of their retention time and compared to a Supelco standard (Supelco 37 Component FAME Mix) and quantified on the basis of multipoint calibration curves. To obtain an estimate of the fatty acid content, we divided the amounts of the respective fatty acids in the extracted samples by the dry weight of the spiderlings used in each sample. The individual dry weight of spiderlings was determined from a subsample of 15 spiderlings from the same egg sac used for the fatty acid analyses, specified beforehand in the fat content section as the initial dry weight.

### 2.4 Statistical Analyses

Statistical analyses were performed with R version 4.3.1 (R Core Team 2023). To assess differences in survival and fat content of spiderlings depending on the winter regime and length of exposure, our explanatory variables were ‘winter regime’ (cold, moderate, or warm) and the two time points of inspection, ‘exposure time’. Other explanatory variables were the clutch size (alive + dead spiderlings + eggs in an egg sac) for survival and the dry weight for fat content. We added the females' ID as a random effect to account for the use of second egg sacs (48%) from one female, specified as (1 Mother ID). We analyzed survival proportion (logit transformed) and fat content using generalized linear mixed models, with the package glmmTMB (Brooks et al. 2017). For the survival proportion, we fitted a model with ordered beta regression (Kubinec 2023) that fits continuous proportion data in the closed interval [0,1]. For the fat content, we used a model that assumed a Tweedie family function, suitable for continuous response variables with zero inflation and over‐dispersion. Model assumptions were checked using the package DHARMa (Hartig and Lohse 2022), and we used a type III ANOVA from the package ‘car’ and a post hoc test with Tukey's correction to test for the significance of the effects of the regimes on response variables with the package emmeans (Lenth et al. 2024). To analyze differences between the means of the different fatty acid groups, an analysis of variance (ANOVA) was applied, followed by a post hoc Tukey HSD test when the assumptions of normality (Shapiro–Wilk test) and equal variances (Levene's test) were met for the majority of fatty acids. When normality assumptions were not met (e.g., for ALA and SDA), a logarithmic data transformation was applied.

### 2.5 RNA Isolation and Sequencing

For each winter regime, we used four replicates each consisting of 15 spiderlings (total *n* = 12). The spiderling pool in each sample was mechanically homogenized using non‐sticky pellet pestles in the presence of 500 μL RNA‐Solv Reagent (Omega Bio‐tek, Norcross, GA, USA), following the manufacturer's recommendations. The isolated RNA was dissolved in 20 μL of nuclease‐free water and quantified using a Qubit 2.0 (Life Technologies). RNA integrity was visualized with a Bioanalyzer 2100 system (Agilent, Santa Clara, CA, USA). Two micrograms of total RNA from each sample was subjected to ribosomal depletion (rRNA) using a custom‐designed *Argiope bruennichi* riboPOOL depletion kit dp‐K096‐000074 (siTOOLs Biotech, Germany) according to the manufacturer's instructions. Ribosomal depleted RNA was purified using the ethanol precipitation option and quantified using the Bioanalyzer 2100 system. We took 100 ng of ribosomal depleted RNA for library construction using the NEBNext Ultra II Directional RNA Library Prep Kit for Illumina (New England Biolabs, Ipswich, MA, USA). Sequencing was done on a NextSeq 500/550 system using a High Output Kit v2.5 flow cell (150 cycles) and 2 × 75 bp paired‐end sequencing.

### 2.6 Differential Gene Expression Analysis

We used fastqc 0.11.9 (http://www.bioinformatics.babraham. ac.uk/projects/fastqc) to check the quality of the raw data. All samples had high‐quality reads and negligible adapter sequence content. The adapter sequences do not interfere, so we proceed with gene mapping. We mapped the reads to the genome of the species (accession No. GCA_947563725.1) (Sheffer et al. 2021) using the program STAR 2.7.11a (Dobin et al. 2013) in two‐pass mode with default parameters. We used the resultant BAM files to generate a count table in Subread 2.0.6, using the featureCounts function (Liao et al. 2014). The count data were normalized with the R package DESq2 1.38.3 (Love et al. 2014) and analyzed via principal components using the plotPCA function. We used the DESq2 function for differential expression analysis, and performed three tests: (1) cold versus moderate, (2) warm versus moderate, and (3) warm versus cold. We compared cold and warm with moderate as a control, and then contrasted gene expression of the warm regime to the cold regime, to find underlying mechanisms of heat and cold tolerance in this recently cold‐adapted population. Transcripts were differentially expressed if the Benjamini and Hochberg adjusted false discovery rate (FDR) *p*‐value was less than 0.05. The overlaps of differentially expressed genes and unique gene sets across the comparisons were calculated and visualized using a VennDiagram package (Chen 2022). To determine whether the overlaps between regime pairs (cold vs. moderate, warm vs. moderate, and warm vs. cold) were greater than would be expected by chance alone, Fisher's exact tests were performed. To test for a difference in count abundance between the genes of interest we used Kruskal‐Wallis tests followed by Dunn post hoc tests.

### 2.7 Functional Enrichment

For each set of differentially expressed genes, a gene ontology (GO) enrichment analysis was performed with the topGO package version 2.60.1 (Alexa and Rahnenfuhrer 2025) to infer the biological function of all differentially expressed genes at an FDR of 0.05. The GO terms were identified in the ontology categories Biological Processes (BP), Molecular Function (MF), and Cellular Component (CC), and the visualization was created with GOplot version 1.0.2 (Walter et al. 2015). Some DEGs of interest were selected based on log_2_ fold‐change strength, high count, identification of probable function (i.e., not identified as uncharacterized loci), and biological relevance related to the GO terms in Table 1. Furthermore, a Kyoto Encyclopedia of Genes and Genomes (KEGG) pathway enrichment analysis (corrected *p* ≤ 0.05) was tested with clusterProfiler version 4.10.0 (Wu et al. 2021) using the *Argiope bruennichi* genome sequence and assembly (Assembly: GCF_947563725.1 chromosome) (Crowley et al. 2023).

## 3 Results

### 3.1 Survival and Fat Content

**FIGURE 2:**
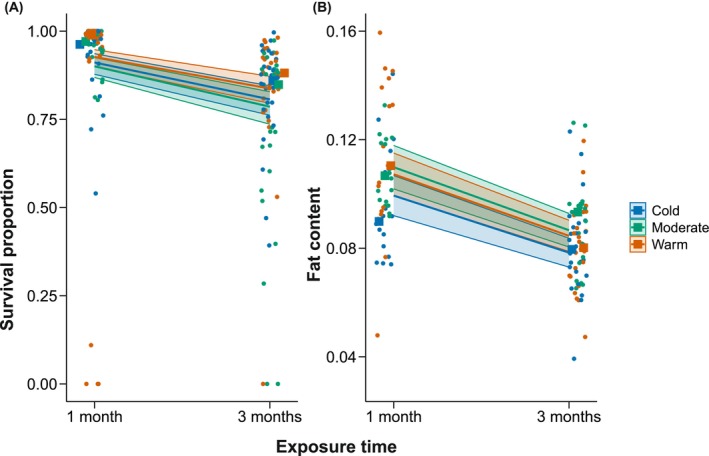
Temperature‐related effects on overwintering *Argiope bruennichi* spiderlings. (A) Proportion of live spiderlings (survival) in egg sacs after one month and three months of exposure to the cold, moderate, and warm winter regimes (B) Fat content of spiderlings after one month and three months of exposure to the winter regimes. Lines represent model predictions flanked by their 95% confidence intervals, points represent data, and squares represent the median of the data.

The proportion of live spiderlings in the egg sacs significantly declined from one month of exposure to the winter regimes (89%) to three months of exposure (78%) (GLMM, χ^2^_1_ = 42.17, *p* < 0.001, Figure 2A). However, there were no significant differences in survival proportion between winter regimes (GLMM, *χ* ^2^_2_ = 3.06, *p* = 0.22, Figure 2A). The clutch size had a significant effect on survival (GLMM, *χ* ^2^_2_ = 5.66, *p* = 0.017), with larger clutches having higher survival proportions; however, this was irrespective of the winter regime. Following the same pattern, the fat content of the spiderlings was overall significantly lower after 3 months (8.3%) compared to 1 month (10.6%) (GLMM, *χ* ^2^_1_ = 47.19, *p* < 0.001, Figure 2B). The spiderlings from different winter regimes did not significantly differ in fat content (GLMM, *χ* ^2^_2_ = 4.45, *p* = 0.11, Figure 2B). The dry weight was significantly related to fat content (GLMM, *χ* ^2^_2_ = 10.66, *p* = 0.001), meaning that for every unit decrease in dry weight, fat proportion decreased by ~13% (Table S2). Therefore, the decrease in weight corresponds to reduced fat reserves.

### 3.2 Fatty Acids

**FIGURE 3:**
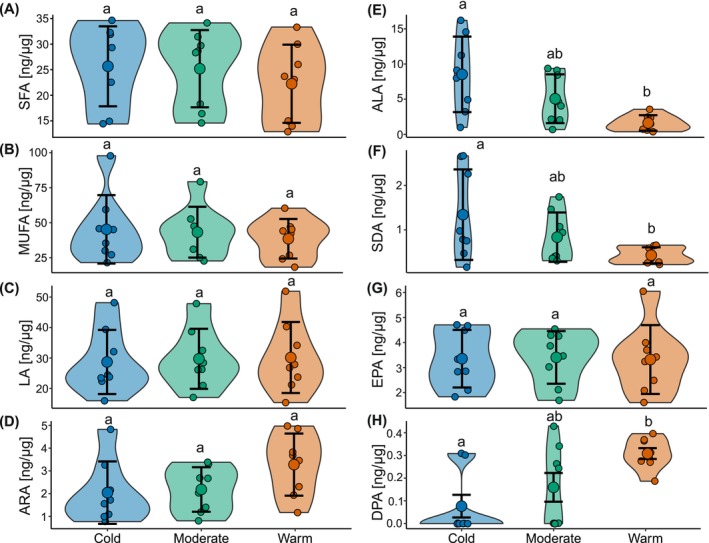
Contents of fatty acid groups and single FA in spiderlings exposed to different winter regimes (cold, moderate, warm) expressed in ng fatty acid per μg body dry weight. (A) Saturated fatty acids (SFA), (B) Monounsaturated fatty acids (MUFA), (C, D) omega‐6 polyunsaturated fatty acids linoleic acid (LA) and arachidonic acid (ARA), respectively, (E–H) omega‐3 PUFAs α‐linolenic acid (ALA), stearidonic acid (SDA), eicosapentaenoic acid (EPA), and docosapentaenoic acid (DPA), respectively. Large circles with error bars represent means ± SD Small circles represent the data points, and the violins represent the distribution of the data. Different lowercase letters indicate significant differences between winter temperature regimes (Tukey HSD post hoc test after ANOVA).

After 3 months of exposure, the spiderlings of the overwintering regimes did not significantly differ in the overall amounts of saturated fatty acids (SFA) and monounsaturated fatty acids (MUFA) (ANOVA, *p* > 0.05; Table S1; Figure 3A,B). Likewise, the ratio between unsaturated and saturated fatty acids did not differ between regimes (ANOVA, F_2,21_ = 0.21, *p* = 0.81; Table S1). However, the spiderlings of the warm winter regime contained 57% less omega‐3 PUFA compared to those of the cold winter regime (Figure S1A, Table S1). This difference was mainly caused by a 5.4‐fold lower content of α‐linolenic acid (ALA, C18:3n‐3; Figure 3E) in the warm compared to the cold winter regime (ANOVA F_2,21_ = 7.40, *p* = 0.004; Table S1), and by stearidonic acid (SDA, C18:4n‐3; Figure 3F; Table S1). While the content of eicosapentaenoic acid (EPA, 20:5n‐3; Figure 3G) was largely constant within the range of 3.4–3.9 ng μg^−1^ in all winter regimes (ANOVA F_2,21_ = 0.01, *p* = 0.99; Table S1), docosapentaenoic acid (DPA, C22:5n‐3, Figure 3H), and docosahexaenoic acid (DHA, C22:6n‐3, Figure S1B), occurred in a higher content in the warm compared to the cold regime (ANOVA F_2,21_ = 4.25, *p* = 0.04; Table S1). The content of omega‐6 PUFAs like linoleic acid (LA, C18:2n‐6; Figure 3C), and arachidonic acid (ARA, 20:4n‐6; Figure 3D) did not differ between regimes (ANOVA F_2,21_ = 2.32, *p* = 0.12; Table S1). The spiderlings in the moderate treatment showed intermediate amounts of the fatty acids that were significantly different between the cold and warm treatments.

### 3.3 Differential Gene Expression Depending on Winter Temperature

**FIGURE 4:**
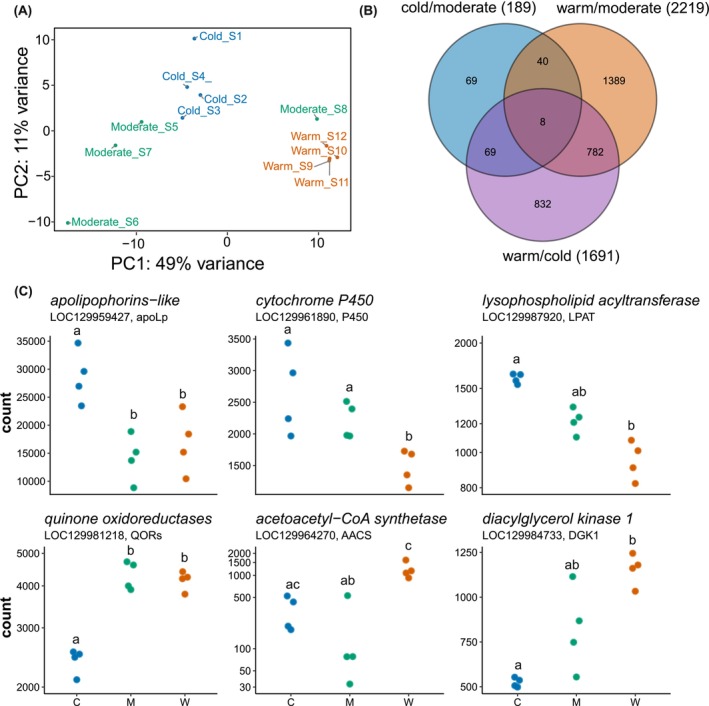
Differential gene expression in *A. bruennichi* spiderlings from three winter regimes cold (C), moderate (M) and warm (W). (A) Principal component analysis on normalized data counts of all differentially expressed genes (DEGs, *n* = 14,893) from 4 RNA‐seq samples per winter regime, each composed of 15 pooled spiderlings: Cold (blue), moderate (green), and warm (orange). (B) Venn diagram showing the number of significant DEGs (FDR < 0.05) when pairs of treatments are compared. (C) Gene counts of the cold regime (blue), moderate (green) and warm (orange) regimes for six example genes highlighting differential abundance between regimes. The top three (*apolipophorins‐like*, *cytochrome P450*, and *lysophospholipid acyltransferase*) show significant differences in gene counts for the cold compared to the warm regime, and the bottom three (*quinone oxidoreductases*, *acetoacetyl‐CoA synthetase*, *diacylglycerol kinase 1*) show significant differences for the warm compared to the cold regime. Different lowercase letters indicate significant differences between regimes (Dunn post hoc test after Kruskal‐Wallis).

We screened for genome‐wide differential gene expression based on RNA‐Seq data. From a total of 37,459 genes, we identified 14,893 differentially expressed genes (DEGs) between the three winter regimes after filtering for low TPM (Transcripts Per Kilobase Million) values and collapsing duplicates. Gene expression patterns were distinctly separated in a principal component space (PC1), with the four samples of cold and warm clustering together, respectively (Figure 4A). More variability is shown in the moderate samples (in both PC1 and PC2), which is also evident in the heat maps, where most of the moderate samples cluster closer to the cold regime and one to the warm regime (Figure S2A,B). We identified 4096 differentially expressed genes (DEGs) with adjusted *p*‐values (padj) < 0.05. In the following, we refer to “upregulated” and “downregulated” genes when the amount of their products was high or low in spiderlings from cold or warm regimes relative to the moderate regime. We compared the spiderlings of the warm relative to the cold regime similarly. The cold regime had 69 unique DEGs (Figure 4B) when compared to the moderate regime (189 in total of which 88 were upregulated, and 101 downregulated). The warm regime had 2219 DEGs in total, 888 of which were upregulated and 1331 downregulated when compared to the moderate regime, and of these 1389 were unique. In comparison to the cold regime, the warm regime had 1691 DEGs in total, 871 of which were upregulated and 820 downregulated, with 832 DEGs being unique. Only 8 DEGs were common to all comparisons (padj < 0.05) (Figure 4B). The warm winter regime showed a higher overall transcriptomic response, as demonstrated by significant effects when comparing warm versus cold and warm versus moderate (Fisher's exact test, two‐tailed *p* < 0.001) (Table S3, Figures S2, S3). The highest contrast in the number of upregulated and downregulated genes with a higher threshold of log_2_ fold change (1 ≥ FC ≤ −1) was found in the warm versus cold comparison, with 156 highly significantly downregulated and 266 significantly upregulated genes (Figures S2C, S3C). Some of the contrasting genes in their expression abundance between cold and warm are shown in Figure 4C: *apolipophorins‐like* (LOC129959427), *cytochrome P450* (LOC129961890), and *lysophospholipid acyltransferase* (LOC129987920) with significant differences in gene counts for cold compared to the warm regime, and *quinone oxidoreductases* (LOC129981218), *acetoacetyl‐CoA synthetase* (LOC129964270), *diacylglycerol kinase 1*(LOC129984733) with significant differences in counts for the warm compared to the cold regime (Table 1).

### 3.4 Functional Enrichment

Significantly enriched gene ontology (GO) terms in the comparison cold versus moderate included structural components of the cytoskeleton, lipid modification, regulation of biological processes, cell communication, signaling and response to stimulus (Figure S4A, Table S4 for the complete list). On the lipid modification GO term, we found two associated upregulated genes *lysophospholipid acyltransferase* (LOC129987920) and *acyl‐CoA dehydrogenase* (LOC129963497) (Table 1, Figure S4A) that play crucial roles in fatty acid metabolism. Additionally, *nose‐resistant fluoxetine* (LOC129962681) and *apolipophorins‐like* (LOC129959427), both involved in lipid transport, were also upregulated. In contrast, there was a relatively strongly downregulated gene in the cell periphery GO term, *adult‐specific rigid cuticular protein* (LOC129968137) (log_2_ fold‐change of −2.83 Table 1).

**TABLE 1:** Selected genes of interest and those identified in the gene ontology (GO) terms from the list of differentially expressed genes (DEGs) for *A. bruennichi* spiderlings. These genes were found in pairwise comparisons between the winter regimes, as specified in the column “comparison.”

Function^a^	Gene name	LOC ID	Symbol	Log_2_fold‐change	Adjusted *p*	Comparison
Cytoskeleton	*Tubulin alpha chain*	LOC129976437	N/A	1.20	7.189E‐03	Cold vs. Moderate
	*Putative ankyrin repeat protein*	LOC129985250	N/A	1.41	7.053E‐09	
Lipid metabolism	*Apolipophorins‐like*	LOC129959427	apoLp	1.02	1.149E‐02	
	*Acyl‐coa dehydrogenase*	LOC129963497	ACAD	0.40	4.497E‐02	
	*Lysophospholipid acyltransferase*	LOC129987920	LPAT	0.39	3.106E‐02	
	*Nose resistant to fluoxetine*	LOC129962681	NRF6	0.90	5.335E‐06	
Mitochondrion	*Mitochondrial Rho gtpase 1*	LOC129971588	Miro1	0.51	5.520E‐02	
Oxidoreductase activity	*Quinone oxidoreductases*	LOC129981218	QORs	−0.84	1.177E‐11	
Chitin‐based extracellular matrix	*Adult‐specific rigid cuticular protein*	LOC129968137	CU24_ARADI	−2.83	5.284E‐03	
Carbohydrate metabolism	*Sucrose‐6‐phosphate hydrolase‐like*	LOC129956957	N/A	1.74	1.244E‐02	Warm vs. Moderate
Lipid metabolism	*Acetoacetyl‐coa synthetase*	LOC129964270	AACS	2.73	7.949E‐03	
	*Fatty acid amide hydrolase 2*	LOC129976441	FAAH2	1.49	3.133E‐05	
	*Diacylglycerol kinase 1*	LOC129984733	DGK1	0.49	4.346E‐02	
	*Phospholipase A2*	LOC129964053	PLA2	−1.41	4.302E‐05	
Heat shock	*Cytochrome P450 3A24‐like*	LOC129957012	P450‐34A24	−1.58	2.170E‐03	
	*Small heat shock protein*	LOC129988413	Hsp20	−1.97	5.919E‐03	
	*Heat shock protein 70 B2*	LOC129958558	Hsp70B2	−2.97	5.179E‐03	
	*Protein lethal(2)essential for life‐like*	LOC129957165	N/A	−2.29	3.819E‐03	
	*Alpha‐crystallin B chain‐like*	LOC129981871	CRYAB	−1.71	6.978E‐04	
	*Alpha‐crystallin A chain‐like*	LOC129984551	CRYAA	−4.20	1.186E‐05	
Purine metabolism	*Adenylosuccinate synthetase*	LOC129983880	AdSS	0.83	2.419E‐03	Warm vs. Cold
Oxidoreductase activity	*Quinone oxidoreductases*	LOC129981218	QORs	0.79	9.527E‐11	
Lipid metabolism	*Low‐density lipoprotein receptor*	LOC129957447	LDLR	1.19	6.205E‐06	
	*Diacylglycerol kinase 1*	LOC129984733	DGK1	1.14	1.916E‐07	
	*Phospholipase A2*	LOC129964053	PLA2	−1.93	2.648E‐10	
	*Lysophospholipid acyltransferase*	LOC129987920	LPAT	−0.74	7.384E‐09	
	*Apolipophorins‐like*	LOC129959427	apoLp	−0.77	2.886E‐02	
Mitochondrion	*Mitochondrial Rho gtpase 1*	LOC129971588	Miro1	−1.02	2.374E‐08	
Signaling	*G‐protein coupled receptor 52‐like*	LOC129963668	GPR52	−1.14	1.102E‐17	
Heat shock	*Cytochrome P450*	LOC129961890	P450	−0.84	9.079E‐04	
	*Small heat shock protein*	LOC129988413	Hsp20	−1.62	2.025E‐02	
	*Alpha‐crystallin A chain‐like*	LOC129984551	CRYAA	−3.41	2.819E‐04	

^a^ The genes were categorized based on their associated functions. Within each category, the genes were ordered by log_2_ fold‐change (a positive value indicates upregulation and a negative value indicates downregulation) and presented with adjusted *p*‐values (padj < 0.05). The gene name and LOC ID were obtained from *Argiope bruennichi* (GCF_947563725.1) genome annotation, and the symbol from UniProt when available.

The warm versus moderate comparison exhibited the highest degree of functional enrichment (Figure S4B, Table S4), in a large number of terms, including those associated with stress responses in general, such as phosphorus metabolic processes, homeostatic processes, phosphorylation, kinase activity and lipoprotein metabolic processes (Table S4, Figure S4B). One gene with a relatively high downregulation (log_2_ fold‐change of −4.20, Table 1) was alpha‐*crystallin A chain‐like* (LOC129984551), which is part of the small heat shock protein family (sHSPs). It prevents protein aggregation under stress conditions together with related proteins such as *alpha‐crystallin B chain‐like* (LOC129981871) (log_2_ fold‐change of −1.71, Table 1), *heat shock protein 70 B2* (LOC129958558), and *protein lethal (2) essential for life‐like* (LOC129957165). Concerning lipid metabolism, we also found *lysophospholipid acyltransferase* (LOC129987920), which was downregulated in the warm and *diacylglycerol kinase 1* (LOC129984733), and *adenylosuccinate synthetase* (LOC129983880) which were upregulated in the warm compared to the moderate treatment (Table 1, Figure S4B).

In the comparison of warm versus cold, enriched GO terms are calcium channel activity, establishment of localization, transport, and peptidase activity—all processes known to be involved in responses to temperature stress (Table S4). Concerning lipid metabolism, *phospholipase A2* (LOC129964053), involved in fatty acid breakdown from phospholipids, is strongly downregulated in the warm compared to the cold regime (log_2_ fold‐change of −1.93; Table 1), whereas the *low‐density lipoprotein receptor* (LOC129957447), which is involved in the uptake of cholesterol‐carrying lipoproteins into the cells, is upregulated (Table 1). The *diacylglycerol kinase 1* (LOC129984733), a gene involved in glycerophospholipid metabolism, had a relatively higher upregulation (log_2_ fold‐change of 1.14; Table 1) in the warm compared to the cold regime.

**FIGURE 5:**
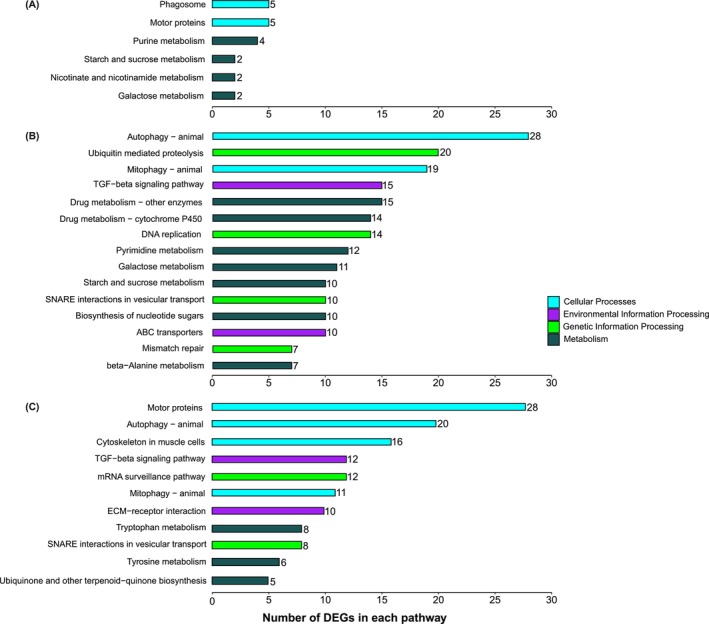
Significant KEGG pathway enrichment in the specific Differentially Expressed Genes (DEGs) (*p*‐value < 0.05) by comparison test in *Argiope bruennichi* spiderlings. (A) cold versus moderate, (B) warm versus moderate, and (C) warm versus cold. The KEGG pathways are listed and ordered according to the number of specific DEGs for each comparison group in each pathway (given on the right side of the bars).

The KEGG enrichment analysis revealed that DEGs could be classified into 39 pathways (FDR < 0.1, Table S5). Significantly enriched pathways (FDR ≤ 0.05) per comparison are shown in Figure 5. KEGG pathways were identified in four categories or Orthology Classes (KEGG orthology classes): Metabolism, Cellular processes, Signaling, and Processing of genetic (or environmental) information. The Metabolism Orthology Class shows the most enriched pathways overall in our regimes (21 of 39), including those involving glycine, serine, and purine metabolism; carbon metabolism; glycerophospholipid metabolism; fatty acid metabolism; galactose metabolism; sphingolipid metabolism; glutathione metabolism; amino acid and nucleotide metabolism; glycerolipid metabolism; starch and sucrose metabolism; nicotinate and nicotinamide metabolism; and tryptophan metabolism. Furthermore, there are other enriched pathways in the processing of genetic and environmental categories, and subcategories like processing in signal transduction, cell differentiation and proliferation, repair proteins, and regulation of calcium concentration (e.g., FOXO signaling pathway, base excision repair, motor proteins, mTOR signaling pathway, and phosphatidylinositol signaling system; Table S5; Figure 5C) that are essential for temperature responses.

## 4 Discussion

We investigated the vulnerability and degree of phenotypic plasticity of a recently cold‐adapted northern population of the wasp spider *Argiope bruennichi* , by exposing overwintering spiderlings to three fixed winter temperature regimes (cold, moderate, and warm) with day/night fluctuations. Survival probability and lipid reserves declined over time, with no difference between winter regimes, demonstrating remarkable tolerance to markedly different temperature regimes in this species. However, the fatty acid composition revealed regime‐specific differences, with omega‐3 polyunsaturated fatty acids reduced under warmer conditions, suggesting elevated metabolic activity during warmer regimes. These physiological responses aligned with variations in gene expression patterns, with the strongest responses observed in the warm regime compared to the other two. Our data suggest that although survival probability and energy usage were similar across the winter regimes, spiderlings experienced physiological stress under the warm winter regime, and showed more targeted responses to the cold winter regime, corroborating local adaptation to the colder environment at the northern edge (Sheffer et al. in press).

We subjected the spiderlings in the egg sacs to challenging fixed winter conditions with strong diurnal temperature fluctuations in the cold and the warm regime, and moderate fluctuations in the third group, and examined survival proportion and fat content over time. Survival proportion and fat reserves declined significantly from one to three months of exposure, suggesting that depletion of lipid stores led to increased mortality. As ectotherms with temperature‐dependent physiological responses (Sinclair 2015), spiders are expected to show similar patterns as insects, in which weight loss during winter correlates with increased mortality and reduced reproductive success (Irwin and Lee Jr. 2003; Knapp and Řeřicha 2020; Nielsen et al. 2022). Weight loss is driven by the depletion of fat reserves, which serve as the primary energy source for overwintering insects (Hahn and Denlinger 2011; Sinclair and Marshall 2018; Anparasan et al. 2025). A steady decline in total lipids was also observed in a cursorial wolf spider species over the course of winter (Potts et al. 2020). In *A. bruennichi* , however, spiderlings are not free‐roaming but confined to the egg sac during winter and unable to replenish energy stores. Consequently, a higher metabolic rate due to challenging winter regimes can incur considerable costs and impact physiology and future survival probability. Such responses are also likely in cursorial spiders, when food availability is limited in winter, but the effects are expected to be more pronounced in *A. bruennichi* , as their spiderlings cannot compensate for depleted energy reserves, and signs of sibling cannibalism inside the egg sacs were not observed.

Research on the role of fatty acid profiles in the overwintering metabolism of spiders is limited. To the best of our knowledge, this study is the first to investigate changes in the fatty acid composition of overwintering spiderlings inside an egg sac. We found a higher ratio of unsaturated to saturated fatty acids in spiderlings of all experimental temperature regimes. This finding is not unexpected, as the temperature dropped below zero each night in all three winter regimes, likely favoring stable unsaturation across treatments. According to Sinclair and Marshall (2018), increased unsaturation of fatty acids is a strategy employed by cold‐adapted insects that prolongs torpor and reduces metabolic rates. This pattern has also been documented in scorpions (Garcia et al. 2021) and spiders (Molina et al. 2024, preprint) under cold conditions.

We observed winter regime‐related differences in the fatty acid composition of the spiderlings. In the cold winter regime, spiderlings contained more omega‐3 PUFAs, with significantly elevated levels of α‐linolenic acid (ALA, C18:3n‐3) compared to the warm winter regime. Omega‐3 PUFAs are characterized by a high degree of unsaturation, possessing multiple double bonds that inhibit tight packing, a feature essential for maintaining membrane fluidity in cold environments (Sinclair and Marshall 2018). Conversely, in warmer environments, membrane fluidity is less important, possibly allowing spiderlings to utilize omega‐3 PUFAs to mitigate temperature‐induced stress. As has been shown before, warmer winter temperatures can elevate metabolic rates and deplete energy stores (Clarke and Fraser 2004; Sinclair 2015; Roberts et al. 2023). Thus, even small temperature changes can have considerable physiological costs as was shown in juveniles of the springtail *Orchesella cincta* (van Dooremalen and Ellers 2010). In contrast, omega‐6 PUFAs remained stable across the temperature regimes in our study. This might be attributed to their smaller effect on membrane fluidity relative to omega‐3 PUFAs (Hazel and Eugene Williams 1990), thereby minimizing the need for temperature‐dependent regulation (Somero et al. 2017).

A mechanism by which spiderlings might modulate their lipid composition in response to winter conditions involves the omega‐3 FA group. Alpha‐linolenic acid (ALA) acts as a precursor for other important omega‐3 FAs, such as eicosapentaenoic acid (20:5n‐3, EPA) and docosahexaenoic acid (22:6n‐3, DHA), both of which are long‐chain fatty acids with known roles in immune function. Similarly, docosapentaenoic acid (DPA, 22:5n‐3) is an intermediate between EPA and DHA, contributing to cell membrane structure and function, and also playing a role in resolving inflammation (Jump 2002). We observed an interesting pattern in these long‐chain omega‐3 PUFAs. Both stearidonic acid (SDA, C18:4n‐3, derivative of ALA) and ALA were present in significantly lower amounts in the warmer winter regime than in the cold regime. In contrast, the levels of DPA and DHA were significantly higher in the warm and lower in the cold regime. The lower levels of SDA and ALA in warmer conditions may indicate their conversion into downstream products such as EPA, DPA, and DHA—all of which are important for immune function and energy transduction (Pilecky et al. 2021). Similar to our findings on temperature‐induced changes in fatty acid composition, recent work on a social spider species that occurs across several climate zones has shown that the chemical composition of the cuticle wax layer changes with acclimation to higher temperatures, thereby mediating thermal tolerance (Malmos et al. 2021).

The gene expression patterns across our temperature regimes showed fewer differentially expressed genes in the cold regime compared to the moderate regime (Figure 4B), especially when contrasted with the other pairwise comparisons. Notably, the moderate regime also experienced nighttime temperatures below 0°C. This likely triggered cold tolerance mechanisms, resulting in a low number of DEGs in this group. Despite the lower number of DEGs in the comparison, there were specific gene expression changes associated with cold hardening. For example, there was an upregulation of genes involved in cytoskeletal dynamics, such as the *tubulin alpha chain* and *putative ankyrin repeat protein* (Table 1), which facilitate the maintenance of proper microtubule function at very low temperatures (Storey and Storey 2013). Cold stress likely also induced the expression of genes involved in lipid metabolism, such as *lysophospholipid acyltransferase*, which is essential for membrane asymmetry (fluidity) and the production of lipid mediator precursors like ARA and EPA (Hishikawa et al. 2008), as well as *acyl‐CoA dehydrogenase*, which is involved in membrane remodeling and fatty acid metabolism (Swigoňová et al. 2009). In contrast, the gene encoding *quinone oxidoreductases*, typically activated under heat stress to mitigate oxidative damage (Lemieux and Blier 2022), was downregulated in the cold regime and expressed at higher levels in both the moderate and warm regimes. This suggests that the moderate regime may also induce temperature stress in *A. bruennichi* , potentially explaining the relatively elevated expression of stress‐response genes in this treatment group (Figure 4A,B).

The chosen temperature regimes were characterized by fluctuating daily temperatures typical of temperate climates, with both the cold and the warm regimes exhibiting a pronounced 20°C difference between day and night, whereas the moderate regime had a daily temperature difference of only 10°C. The spiderlings in the cold and warm regimes showed more specific responses to temperature stress, suggesting that strong daily fluctuations elicit more targeted responses in gene expression than the moderate regime. Williams, Hellmann, and Sinclair (2012); Williams, Marshall, et al. (2012) showed that overwintering butterfly pupae performed better under high daily fluctuation compared to a constant temperature. Furthermore, high daily temperature fluctuations can alter the optimal and critical maximum temperatures, as well as the thermal reaction norm of a species (Paaijmans et al. 2013). For example, artic mites and springtails (cold‐adapted) lowered their upper thermal limits when acclimated to “moderate” temperatures that lacked high daily fluctuations (Sørensen et al. 2024). The *A. bruennichi* spiderlings that were subjected to the moderate regime displayed intermediate or equal values when compared to the other regimes in survival, fat content, and fatty acid content, but showed high variation in the gene expression response. This pattern suggests that milder daily thermal variation may not impose strong directional selection on physiological traits but instead elicits more variable molecular responses, indicating that the amplitude of daily temperature fluctuations plays a key role in the specificity of thermal responses in *A. bruennichi* spiderlings.

When comparing warm and moderate winter regimes, we identified general stress responses involving phosphorus metabolic processes, homeostatic processes, and the regulation of biological processes. Additional stress responses with high gene involvement included transferase activity and adenyl nucleotide binding (ATP binding), consistent with the findings of a transcriptomic study on a spider species subjected to high‐temperature stress (Chen et al. 2023). Regarding lipid metabolism, *acetoacetyl‐CoA synthetase*—a key enzyme in ketone body (KB) metabolism—showed high relative expression in both the warm and cold regimes compared to the moderate regime. This enzyme facilitates the conversion of acetoacetate, a ketone body, to acetoacetyl‐CoA, which is used in lipogenesis and cholesterol synthesis (Bergstrom 2023). The elevated gene expression in the warm regime suggests increased lipogenesis, while in the cold regime, the higher activity of *acyl‐CoA dehydrogenase* indicates enhanced fatty acid catabolism to support membrane remodeling and fluidity. Krehenwinkel et al. (2015) reported a similar pattern from northern populations of *A. bruennichi* under cold stress, with increased acetoacetate‐CoA ligase activity. Interestingly, *acyl‐CoA dehydrogenase* was found to be downregulated in bumblebees subjected to acute cold exposure at 0°C (Verble et al. 2024), suggesting that its role becomes more prominent below this threshold. Other genes involved in lipid metabolism, such as *fatty acid amide hydrolase 2*—which regulates fatty acid signaling—and *diacylglycerol kinase 1*, were more abundant in the warm than in the cold regime. The latter produces diacylglycerol (DAG), a critical lipid intermediate in lipid biosynthetic pathways that can function as a signaling lipid (Shulga et al. 2011).

Comparing the warm and cold winter regimes, we found small heat shock proteins and related genes. Expression was lower in the warm regime but higher in the moderate and cold regimes (Table 1, Figure S3B,C). Previous studies on *A. bruennichi* and a damselfly (*Ischnura elegans*) have documented increased cold tolerance during range expansions into colder regions, with no change in heat tolerance and even downregulation of genes involved in heat shock responses, likely due to relaxed selection on heat tolerance in colder environments (Krehenwinkel and Tautz 2013; Lancaster 2016). On the other hand, Lancaster (2016) reported that several genes upregulated in response to heat stress in core populations were also upregulated in response to cold stress in edge populations. This is likely selectively favored, as heat shock proteins can prevent protein denaturation at cold temperatures by assisting in folding and establishing the correct protein conformation under various stress conditions (Teets and Denlinger 2013).

Further genes exhibiting high relative expression in the cold regime included *apolipophorins‐like* (ApoLp), which are responsible for lipid transport between tissues and play a role in modulating cellular responses and immunity (Wrońska et al. 2023). This pattern was accompanied by elevated expression of *Phospholipase A2*, an enzyme that hydrolyzes phospholipids to release arachidonic acid (ARA) and other fatty acids from cell membranes, as well as *lysophospholipid transferase*, as previously mentioned. Together, these enzymes contribute to the maintenance of membrane asymmetry and facilitate the release of key signaling precursors. The upregulation of these lipid‐metabolizing enzymes under cold conditions suggests enhanced mobilization and remodeling of lipids, which is consistent with previous findings that cold exposure generally promotes fatty acid oxidation in animals (Lemieux and Blier 2022). In contrast to this cold‐induced upregulation of lipid catabolism, the *low‐density lipoprotein receptor*, which mediates the uptake of cholesterol‐carrying lipoproteins into cells, showed higher expression in the warm regime (and lower in the cold regime). This may reflect an increased demand for cholesterol in membrane synthesis or signaling under heat stress. The regulation of genes involved in lipid transport may also reflect the freeze‐avoidant strategy of *A. bruennichi*, which produces cryoprotectants and antifreeze proteins that lower its freezing point (on average −28.75°C in northern spiderlings) (Sheffer et al. in press).

The gene ontology analysis of the differential gene expression datasets indicates that the majority of genes are involved in metabolic processes (Table S4), a common finding in both cold and heat resistance studies in insects (Sinclair 2015; Mikucki and Lockwood 2021). We can infer that the spiderlings of *A. bruennichi* predominantly utilize lipids to maintain adequate membrane fluidity and functionality in the cold regime. In contrast, in the warm regime, carbohydrates and amino acids seem to serve as the primary energy sources, as evidenced by GO terms like phosphorylation and peptidase activity, along with the associated upregulated genes such as *sucrose‐6‐phosphate hydrolase‐like* and *adenylosuccinate synthetase*. The utilization of lipids in the warm winter regime is more likely directed toward immune response, corroborating our general body lipid analyzes, which demonstrated a decrease in lipid storage during winter across the regimes (Figure 2B). These findings also correlate with our fatty acids data, which revealed the differential allocation of lipid reserves by spiderlings depending on the winter regime. The differential use of fatty acids underscores the intricate balance that overwintering spiderlings must maintain for survival. It also emphasizes the potential effects of environmental changes on their lipid dynamics, which are crucial for growth, reproduction and survival (Twining et al. 2021). Spiderlings disperse shortly after leaving the egg sac by ballooning (Walter et al. 2005) and must construct their first web to capture prey and proceed with development by molting several times until adulthood. If energy reserves are depleted after a metabolically demanding warm winter, web construction, dispersal capacity, and subsequent survival may be compromised.

The Kyoto Encyclopedia of Genes and Genomes (KEGG) analysis further corroborates that metabolic pathways, including those involved in carbon, amino acid, nucleic acid, and fatty acid metabolism, were the most enriched responses of spiderlings to our winter regimes. The findings indicate that the DEGs exhibit distinct pathway enrichment depending on the exposure temperature. For instance, the level of purine metabolism, which is essential for energy transfer (e.g., via ATP and GTP) and effective cell signaling, was lower in the cold regime, indicating lower metabolic activity compared to the warm regime. Similarly, pathways such as nicotinate and nicotinamide metabolism, tryptophan metabolism, starch and sucrose metabolism, and galactose metabolism were also downregulated in the cold regime. These findings are consistent with the patterns observed in cold‐adapted Antarctic insects, which suppress certain metabolic processes to prioritize cryoprotection over hydration (Teets et al. 2012). Additionally, pyrimidine metabolism, which provides key components for RNA and phospholipid synthesis, and ubiquinone biosynthesis, crucial for cellular energy production and antioxidative defense, were among the enriched pathways in the warm treatment. These pathways are commonly involved in temperature stress responses (Teets and Denlinger 2013; Chen et al. 2023) and have been shown to be upregulated in organisms such as fire ants following heat exposure (Vatanparast et al. 2021).

While our KEGG analysis primarily focused on metabolic pathways directly linked to temperature responses, we also detected significant changes in other cellular and genetic processes. Certain pathways were consistently observed across the comparisons of treatments, highlighting their potential importance in coping with winter temperature variations. For example, SNARE interactions in vesicular transport were significantly enriched in both comparisons involving the warm regime. As SNARE proteins mediate membrane fusion and vesicular transport, their increased enrichment suggests increased activity of these processes under warmer conditions. In contrast, pathways related to motor protein activity and cytoskeletal components in muscle cells were consistently enriched in the cold regime. These findings are consistent with previously reported cold‐induced cytoskeletal reorganization in insects (Teets et al. 2012) and spider mites (Bryon et al. 2017), indicating that *A. bruennichi* spiderlings may employ a similar strategy to maintain cellular integrity under cold stress.

When species expand their range, they often retain the phenotypic plasticity that evolved in their ancestral environments (Ho et al. 2020). Accordingly, the observed responses of *A. bruennichi* spiderlings to diverse winter temperature regimes can be considered in the context of the recent northward range expansion of the species. In a reciprocal common garden experiment, a suite of cold tolerance traits was identified that showed not only phenotypic plasticity and signatures of local adaptation in northern populations, but also a surprisingly high cold tolerance in spiderlings from southern populations, which might have contributed to the range expansion success (Sheffer et al. in press). Ongoing investigations on the potential and limits of local adaptation and phenotypic plasticity in *A. bruennichi* focus on the underlying molecular and physiological mechanisms that facilitated the expansion of the species. Furthermore, we investigate if increasingly warmer winters will affect survival, metabolism, and temperature tolerance in the core region of the species, thereby leading to a northward range shift.

Our study elucidates the performance of *A. bruennichi* and the molecular and physiological mechanisms underlying thermal tolerance at its northern range edge. The ability to modulate fatty acid composition and the expression of genes from metabolic pathways under different winter regimes likely reflects the potential to cope with rapidly changing environmental conditions through phenotypic plasticity. Because spiders from southern and northern populations are clearly genetically differentiated and show significant genotype‐environment associations (Krehenwinkel and Tautz 2013; Sheffer et al. in press), we expect that the population used in our study is representative of the northern region. Given the broad reaction norm that we discovered, *A. bruennichi* is able to survive very different winter regimes. They are likely to cope with severely cold spells during winter, resulting from reduced snow cover due to climate warming, as well as with relatively warm winters. However, such plastic adjustments may entail metabolic costs that can affect post‐winter survival and growth, which requires further investigation. Our study provides insights into the degree of plasticity in response to controlled winter regimes. In a subsequent reciprocal common garden experiment, we will explore differences in gene expression dynamics between spiderlings from the core and edge of the distribution and under temperature regimes that simulate the natural course during winter. In conclusion, our research expands our knowledge of how spiders are affected by winter conditions and highlights the importance of considering survival, physiological performance, and molecular responses when assessing species' vulnerability or resilience to climate change.

## Author Contributions

**Carolina Ortiz‐Movliav:** conceptualization (equal), data curation (lead), formal analysis (lead), investigation (lead), methodology (equal), project administration (lead), validation (equal), visualization (lead), writing – original draft (lead), writing – review and editing (lead). **Marina Wolz:** conceptualization (supporting), data curation (supporting), formal analysis (supporting), investigation (supporting), methodology (supporting), writing – review and editing (supporting). **Michael Klockmann:** conceptualization (equal), formal analysis (supporting), investigation (equal), methodology (supporting), project administration (supporting), writing – review and editing (supporting). **Andreas Walter Kuss:** methodology (supporting), resources (supporting), software (supporting), supervision (equal), validation (supporting), writing – review and editing (supporting). **Lars Riff Jensen:** conceptualization (supporting), methodology (supporting), resources (supporting), software (supporting), supervision (supporting), validation (supporting), writing – review and editing (supporting). **Corinna Jensen:** methodology (supporting), software (supporting), supervision (supporting), validation (supporting), writing – review and editing (supporting). **Alexander Wacker:** data curation (supporting), formal analysis (supporting), investigation (supporting), methodology (supporting), resources (equal), software (equal), supervision (equal), validation (equal), visualization (supporting), writing – review and editing (equal). **Gabriele B. Uhl:** conceptualization (equal), funding acquisition (lead), investigation (equal), methodology (equal), project administration (supporting), resources (equal), software (equal), supervision (lead), validation (equal), visualization (supporting), writing – original draft (supporting), writing – review and editing (equal).

## Conflicts of Interest

The authors declare no conflicts of interest.

## Supporting information


**Data S1:** ece372507‐sup‐0001‐Supinfo.pdf.

## Data Availability

The sequencing data are available on NCBI's Gene Expression Omnibus and is accessible through GEO Series accession number GSE287559. R and bash scripts of data analysis and datasets are available on GitHub: https://github.com/CarolinaOrtizM/Estonia‐RNAseq.
