# Supplementary material for: Winter Temperature Affects Fatty Acid Composition and Gene Expression, but Not Fat Content and Survival in a Northern Population of a Range‐Expanding Spider
Source: Ecol Evol. 2025 Nov 27;15(12):e72507. doi: 10.1002/ece3.72507 (PMC12658614; doi:10.1002/ece3.72507)
Supplement: Supplementary file 1 — Data S1: ece372507‐sup‐0001‐Supinfo.pdf. [file ECE3-15-e72507-s001.pdf]

Supplement

Winter temperature affects fatty acid composition and gene expression, but not fat content and survival in a northern population of a range-expanding spider

Table S1. Fatty acids contents (mean ± SE; ng per µg of dry mass) of the spiderlings from the three winter regimes. Only the fatty acids accounting for > 1% of total fatty acids in at least one sample are shown. Σ SFAs is the sum of all saturated fatty acids. Σ MUFAs is the sum of all monounsaturated fatty acids. Σ ω-3 PUFAs is the sum of all polyunsaturated omega-3 fatty acids, and Σ ω-6 PUFAs is the sum of all polyunsaturated omega-6 fatty acids. U/S is the ratio of unsaturated over saturated fatty acids. Table shows F- and p-values of ANOVA, and p-values of multiple comparisons among means of the three different treatments Tukey HSD tests. Statistically significant values (<0.05) of ANOVA and Tukey HSD tests are depicted in bold, red font.

	**Winter regimes**					**Tukey HSD**		
**Fatty acid**	**Cold(c)**	**Moderate(m)**	**Warm(w)**	$F_{2,21}$	**ANOVA p**	**m -c**	**w- c**	**w- m**
C16:0	8.15 ± 3.27	7.97 ± 3.42	6.75 ± 2.00	0.527	0.598	0.992	0.620	0.692
C18:0	16.12 ± 4.33	15.87 ± 3.87	13.77 ± 4.71	0.714	0.501	0.992	0.532	0.601
C20:0	0.59 ± 0.42	0.52 ± 0.34	0.49 ± 0.39	0.122	0.886	0.934	0.883	0.992
C22:0	0.81 ± 0.36	0.83 ± 0.55	1.24 ± 1.00	0.970	0.396	0.998	0.448	0.479
C24:0	0.09 ± 0.17	0.15 ± 0.20	0.09 ± 0.16	0.281	0.758	0.806	0.999	0.784
**Σ SFA**	25.66 ± 7.81	25.20 ± 7.54	22.26 ± 7.63	0.465	0.635	0.992	0.653	0.727
C16:1n-9	1.19 ± 1.62	0.64 ± 0.66	1.02 ± 1.25	0.423	0.661	0.645	0.953	0.818
C16:1n-7	1.07 ± 0.74	1.12 ± 0.65	1.31 ± 0.90	0.209	0.813	0.990	0.814	0.881
C18:1n12	3.22 ± 2.18	5.41 ± 6.64	3.89 ± 3.11	0.521	0.601	0.587	0.949	0.772
C18:1n-9	38.97 ± 22.02	34.96 ± 20.90	31.42 ± 10.99	0.328	0.724	0.903	0.702	0.924
C20:1n-9	0.22 ± 0.32	0.33 ± 0.31	0.09 ± 0.11	1.631	0.220	0.699	0.590	0.193
C22:1n-9	0.32 ± 0.35	0.50 ± 0.65	0.47 ± 0.22	0.364	0.699	0.707	0.790	0.989
C24:1	0.25 ± 0.24	0.28 ± 0.22	0.33 ± 0.10	0.307	0.739	0.968	0.727	0.860
**Σ MUFA**	45.26 ± 24.46	43.24 ± 18.15	38.54 ± 14.14	0.253	0.779	0.977	0.769	0.879
C18:4 n-3 (SDA)	1.34 ± 1.02	0.83 ± 0.56	0.43 ± 0.17	3.636	**0.044**	0.314	**0.035**	0.466
C18:3 n-3 (ALA)	8.53 ± 5.37	5.06 ± 3.47	1.62 ± 1.10	7.402	**0.004**	0.358	**0.003**	0.064
C20:3 n-3	0.28 ± 0.30	0.41 ± 0.84	0.28 ± 0.26	0.168	0.847	0.869	0.999	0.873
C20:5 n-3 (EPA)	3.35 ± 1.15	3.41 ± 1.05	3.32 ± 1.37	0.010	0.990	0.100	0.972	0.976

C20:4 n-3	0.41 ± 0.49	0.23 ± 0.25	0.32 ± 0.31	0.510	0.608	0.579	0.870	0.869
C22: 5 n-3 (DPA)	0.08 ± 0.14	0.16 ± 0.18	0.31 ± 0.07	5.841	**0.010**	0.462	**0.008**	0.100
C22:6 n-3 (DHA)	0.04 ± 0.07	0	0.22 ± 0.21	6.237	**0.008**	0.829	**0.003**	**0.009**
**Σ ω-3 PUFA**	14.12 ± 7.15	10.11 ± 4.87	6.49 ± 2.69	4.254	**0.038**	0.452	**0.030**	0.295
C18:2 n-6 (LA)	28.69 ± 10.53	29.73 ± 9.85	30.16 ± 11.65	0.039	0.961	0.979	0.959	0.996
C18:3 n-6	0.53 ± 0.52	0.46 ± 0.41	0.85 ± 1.06	0.662	0.526	0.974	0.664	0.532
C20:2 n-6	0.05 ± 0.07	0.03 ± 0.04	0.04 ± 0.04	0.281	0.758	0.737	0.935	0.916
C20:3 n-6	0.22 ± 0.43	0.22 ± 0.26	0.23 ± 0.23	0.002	0.998	0.999	0.998	0.999
C20:4 n-6 (ARA)	2.05 ± 1.37	2.18 ± 0.98	3.28 ± 1.36	2.322	0.123	0.973	0.145	0.212
C22:2 n-6	0.44 ± 0.52	0.47 ± 0.47	0.47 ± 0.55	0.007	0.993	0.994	0.994	0.999
**Σ ω-6 PUFA**	31.98 ± 12.36	33.09 ± 10.69	35.02 ± 13.38	0.127	0.881	0.982	0.873	0.946
**U/S**	3.6	3.4	3.6	0.438	0.651	0.826	0.941	0.632
**TOTAL FA**	117.02 ± 37.63	111.63 ± 33.13	102.30 ± 33.49	0.366	0.698	0.948	0.679	0.855

Table S2. Results of generalized linear mixed models testing for the effect of regime and opening (exposure time) on the response variables for *A. bruennichi* spiderlings subjected to three winter regimes. Bold p-values indicate statistically significant (<0.05) differences.

Response variable	N	Fixed effects	Estimate	Chi^2	*df*	*p*
Survival proportion	152	**Opening**	-1.007	42.17	1	**8.36E-11**
		Regime	0.215	3.05	2	0.217
		**Clutch size***	0.179	5.66	1	**0.017**
Fat content	125	**Opening**	-0.246	47.19	1	**6.45E-12**
		Regime	0.056	4.45	2	0.108
		**Dry weight**	0.138	10.66	1	**0.001**

*scaled in the model

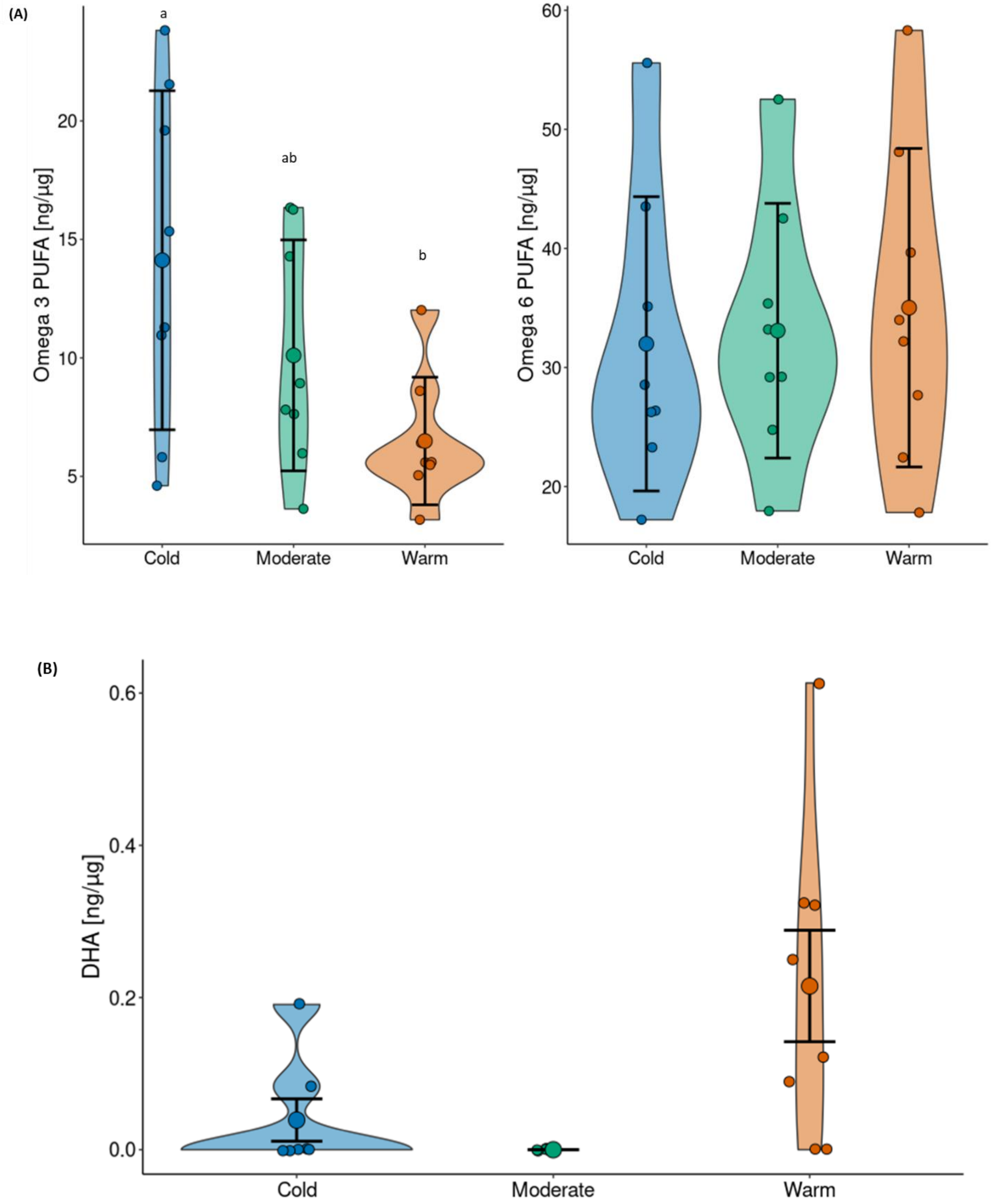

Figure S1. Amounts of polyunsaturated fatty acid groups in spiderlings according to winter regimes (cold, moderate, warm) (in ng fatty acid by µg of dry weight per individual) **(A)** Omega-3 PUFAs (left) and

Omega-6 PUFAs (right), and **(B)** Docosahexaenoic acid (DHA) a long-chained omega-3-PUFA. Large circles with error bars represent means ± s.d. Small circles represent the data points, and the violins represent the distribution of the data. Different lowercase letters indicate significant differences between regimes (Tukey HSD post hoc test after ANOVA).

Table S3. Fisher's Exact Test for differentially gene expressed (DGE) data.

	**Odds ratio**	**p-value**
cold vs moderate	1.11538	0.6121
warm vs cold	0.65566	0.0068
warm vs moderate	0.03801	0.00001

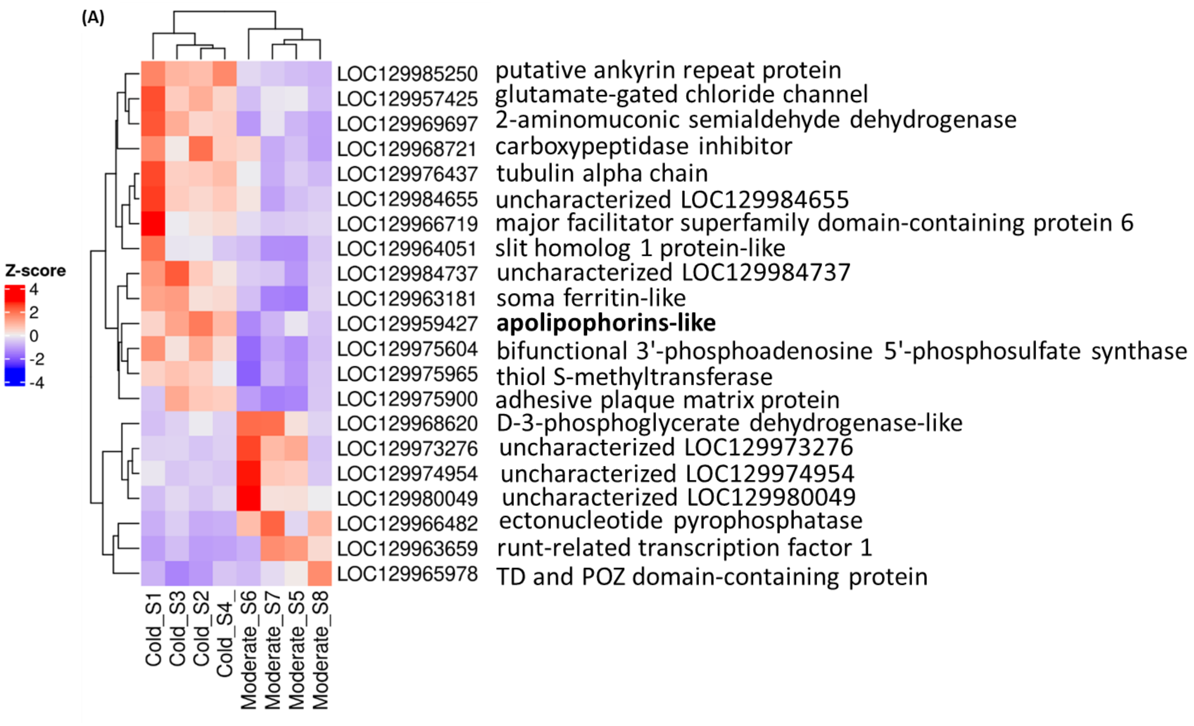

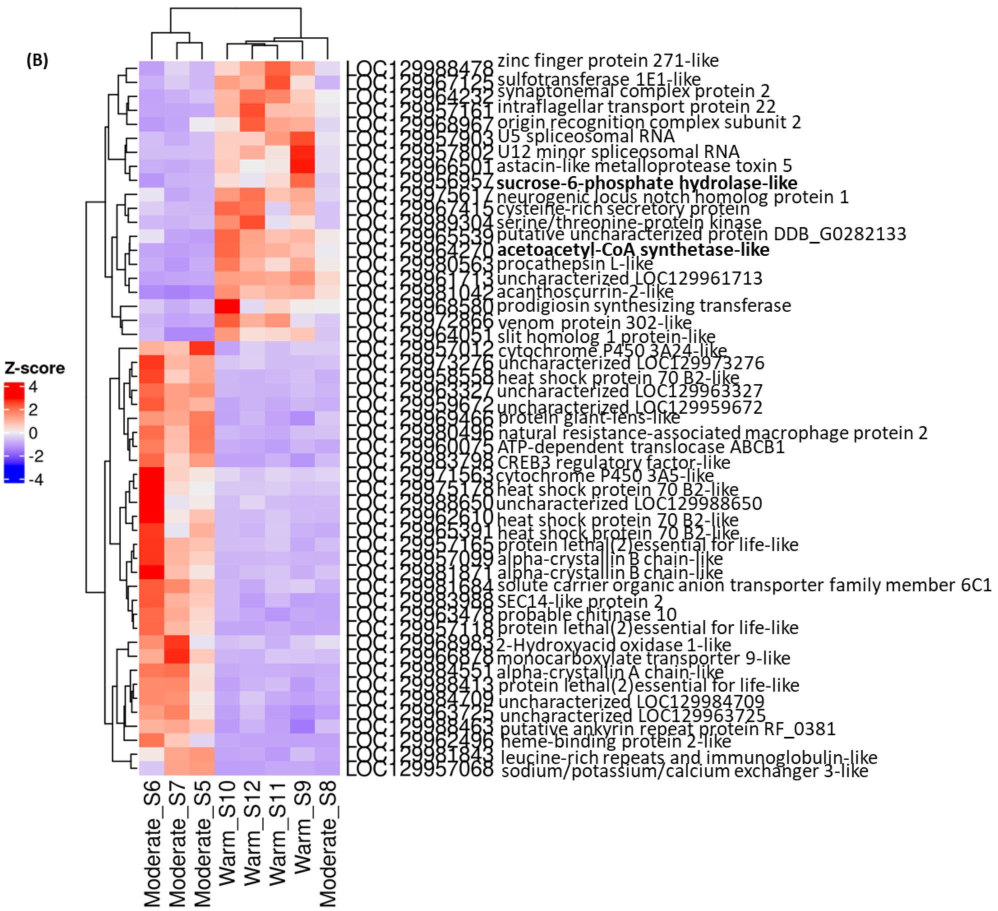
(B)
Z-score
4
2
0
-2
-4
LOC129988478 zinc finger protein 271-like
LOC129967125 sulfotransferase 1E1-like
LOC129964232 synaptonemal complex protein 2
LOC129957161 intraflagellar transport protein 22
LOC129968967 origin recognition complex subunit 2
LOC129957903 U5 spliceosomal RNA
LOC129957802 U12 minor spliceosomal RNA
LOC129966501 astacin-like metalloprotease toxin 5
LOC129956957 sucrose-6-phosphate hydrolase-like
LOC129975617 neurogenic locus notch homolog protein 1
LOC129967415 cysteine-rich secretory protein
LOC129989304 serine/threonine-protein kinase
LOC129965539 putative uncharacterized protein DDB_G0282133
LOC129964270 acetoacetyl-CoA synthetase-like
LOC129980563 procathepsin L-like
LOC129961713 uncharacterized LOC129961713
LOC129981042 acanthoscurrin-2-like
LOC129968580 prodigiosin synthesizing transferase
LOC129972866 venom protein 302-like
LOC129964051 slit homolog 1 protein-like
LOC129957012 cytochrome P450 3A24-like
LOC129973276 uncharacterized LOC129973276
LOC129958558 heat shock protein 70 B2-like
LOC129963327 uncharacterized LOC129963327
LOC129959672 uncharacterized LOC129959672
LOC129969466 protein giant-lens-like
LOC129980496 natural resistance-associated macrophage protein 2
LOC129960075 ATP-dependent translocase ABCB1
LOC129983798 CREB3 regulatory factor-like
LOC129971563 cytochrome P450 3A5-like
LOC129975178 heat shock protein 70 B2-like
LOC129988650 uncharacterized LOC129988650
LOC129962610 heat shock protein 70 B2-like
LOC129965391 heat shock protein 70 B2-like
LOC129957165 protein lethal(2)essential for life-like
LOC129957099 alpha-crystallin B chain-like
LOC129981871 alpha-crystallin B chain-like
LOC129981684 solute carrier organic anion transporter family member 6C1
LOC129983988 SEC14-like protein 2
LOC129963478 probable chitinase 10
LOC129957118 protein lethal(2)essential for life-like
LOC129968983 2-Hydroxyacid oxidase 1-like
LOC129966878 monocarboxylate transporter 9-like
LOC129984551 alpha-crystallin A chain-like
LOC129988413 protein lethal(2)essential for life-like
LOC129984709 uncharacterized LOC129984709
LOC129963725 uncharacterized LOC129963725
LOC129988463 putative ankyrin repeat protein RF_0381
LOC129962496 heme-binding protein 2-like
LOC129981843 leucine-rich repeats and immunoglobulin-like
LOC129957068 sodium/potassium/calcium exchanger 3-like
Moderate_S6
Moderate_S7
Moderate_S5
Warm_S10
Warm_S12
Warm_S11
Warm_S9
Moderate_S8

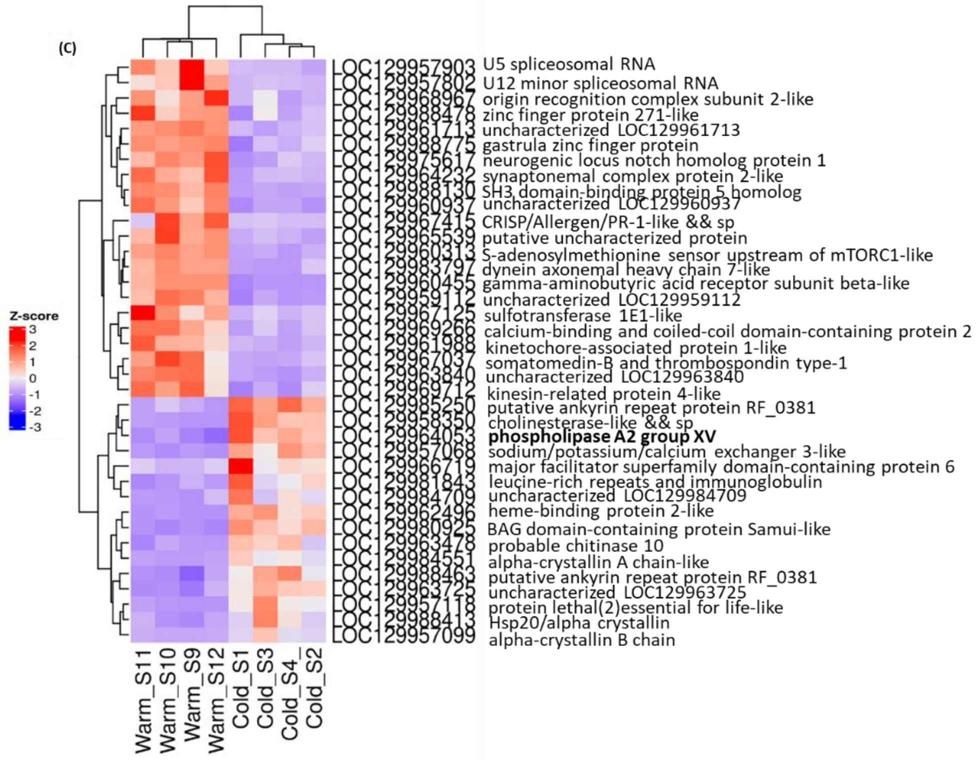
(C)
Z-score
3
2
1
0
-1
-2
-3
LOC129957903 U5 spliceosomal RNA
LOC129957802 U12 minor spliceosomal RNA
LOC129968967 origin recognition complex subunit 2-like
LOC129988478 zinc finger protein 271-like
LOC129961713 uncharacterized LOC129961713
LOC129988775 gastrula zinc finger protein
LOC129975617 neurogenic locus notch homolog protein 1
LOC129964232 synaptonemal complex protein 2-like
LOC129988130 SH3 domain-binding protein 5 homolog
LOC129960937 uncharacterized LOC129960937
LOC129967415 CRISP/Allergen/PR-1-like && sp
LOC129965539 putative uncharacterized protein
LOC129960313 S-adenosylmethionine sensor upstream of mTORC1-like
LOC129983797 dynein axonemal heavy chain 7-like
LOC129960455 gamma-aminobutyric acid receptor subunit beta-like
LOC129959112 uncharacterized LOC129959112
LOC129967125 sulfotransferase 1E1-like
LOC129969268 calcium-binding and coiled-coil domain-containing protein 2
LOC129961988 kinetochore-associated protein 1-like
LOC129967037 somatomedin-B and thrombospondin type-1
LOC129963840 uncharacterized LOC129963840
LOC129969712 kinesin-related protein 4-like
LOC129985250 putative ankyrin repeat protein RF_0381
LOC129958350 cholinesterase-like && sp
LOC129964053 phospholipase A2 group XV
LOC129957068 sodium/potassium/calcium exchanger 3-like
LOC129966719 major facilitator superfamily domain-containing protein 6
LOC129981843 leucine-rich repeats and immunoglobulin
LOC129984709 uncharacterized LOC129984709
LOC129962496 heme-binding protein 2-like
LOC129980925 BAG domain-containing protein Samui-like
LOC129963478 probable chitinase 10
LOC129984551 alpha-crystallin A chain-like
LOC129988463 putative ankyrin repeat protein RF_0381
LOC129963725 uncharacterized LOC129963725
LOC129957118 protein lethal(2)essential for life-like
LOC129988413 Hsp20/alpha crystallin
LOC129957099 alpha-crystallin B chain
Warm_S11
Warm_S10
Warm_S9
Warm_S12
Cold_S1
Cold_S3
Cold_S4_
Cold_S2

Figure S2. Heatmaps of normalized gene expression values of significant DEGs (-1.5 < FC > 1.5; padj < 0.05). (A) cold vs moderate, **(B)** warm vs moderate and **(C)** warm vs cold. The Z-score represents the normalized gene expression value. Red indicates upregulated expression and blue indicates downregulated expression. Columns correspond to samples and the rows to genes.

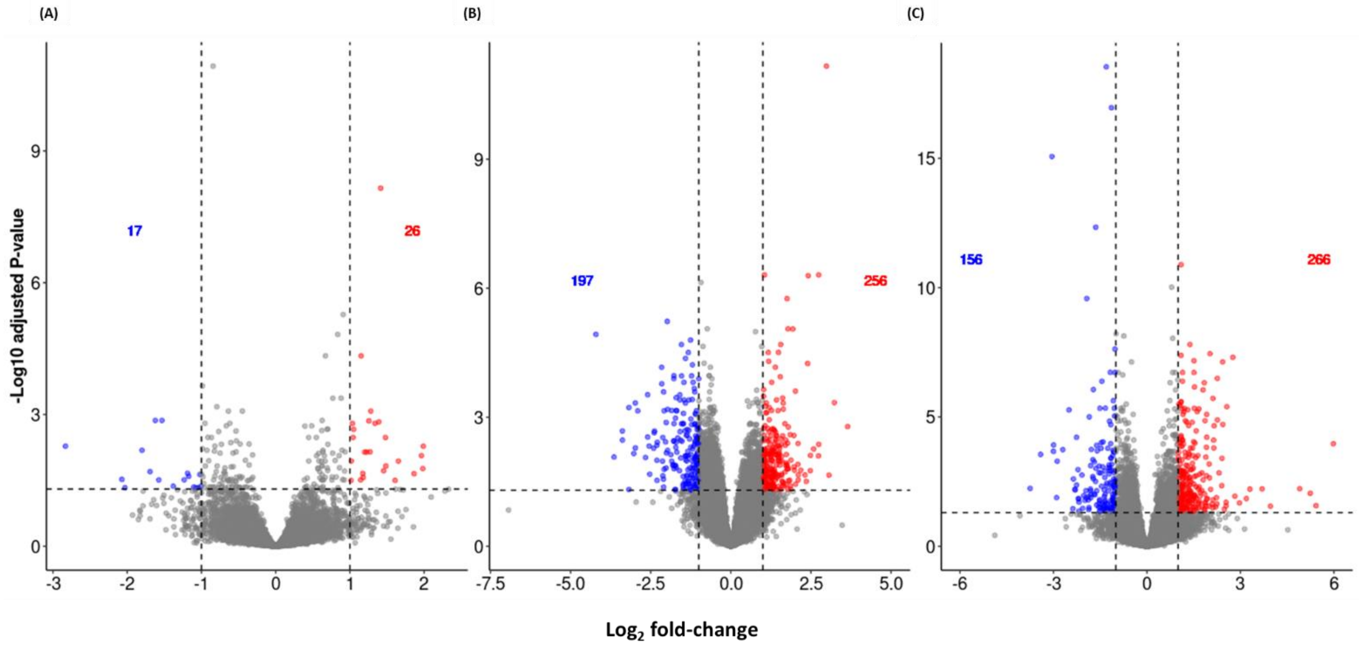

Figure S3. Volcano plots of significantly DEGs with log2FC > 1 and log2FC < -1 (A) cold vs moderate, **(B)** warm vs moderate and **(C)** warm vs cold. No significant genes are represented in grey. Significantly(p-adj < 0.05) upregulated genes are shown in red and downregulated genes in blue.

Table S4. Significantly enriched Gene Ontology (GO) categories for differentially expressed genes in the cold, moderate or warm winter treatments at a false detection rate (FDR) of 0.05. # refers to the number of enriched transcripts in the category. BP = Biological processes, MF= Molecular function and CC = cellular component.

GO ID	GO category	Term	#	Comparison
GO:0023052	signaling	BP	28	cold vs moderate
GO:0007154	cell communication	BP	28	
GO:0030258	lipid modification	BP	3	
GO:0006303	double-strand break repair via nonhomologous	BP	2	
GO:0015267	channel activity	MF	9	
GO:0005200	structural constituent of cytoskeleton	MF	5	
GO:0005254	chloride channel activity	MF	4	
GO:0099081	supramolecular polymer	CC	7	

GO:0043412	macromolecule modification	BP	238	warm vs moderate
GO:0006793	phosphorus metabolic process	BP	180	
GO:0006796	phosphate-containing compound metabolic process	BP	179	
GO:0048519	negative regulation of biological process	BP	105	
GO:0022402	cell cycle process	BP	65	
GO:0042592	homeostatic process	BP	34	
GO:0046486	glycerolipid metabolic process	BP	29	
GO:0042157	lipoprotein metabolic process	BP	17	
GO:0043543	protein acylation	BP	15	
GO:0042158	lipoprotein biosynthetic process	BP	13	
GO:0016740	transferase activity	MF	249	
GO:0030554	adenyl nucleotide binding	MF	199	
GO:0005524	ATP binding	MF	178	
GO:0004674	protein serine/threonine kinase activity	MF	40	
GO:0032993	protein-DNA complex	CC	43	
GO:0005730	nucleolus	CC	23	
GO:0051234	establishment of localization	BP	224	warm vs cold
GO:0006810	transport	BP	219	
GO:0097190	apoptotic signaling pathway	BP	6	
GO:0008233	peptidase activity	MF	60	
GO:0005216	monoatomic ion channel activity	MF	44	
GO:0017171	serine hydrolase activity	MF	19	
GO:0015085	calcium ion transmembrane transporter	MF	12	
GO:0005262	calcium channel activity	MF	10	
GO:0000030	mannosyltransferase activity	MF	9	
GO:0005739	mitochondrion	CC	70	
GO:0098797	plasma membrane protein complex	CC	22	

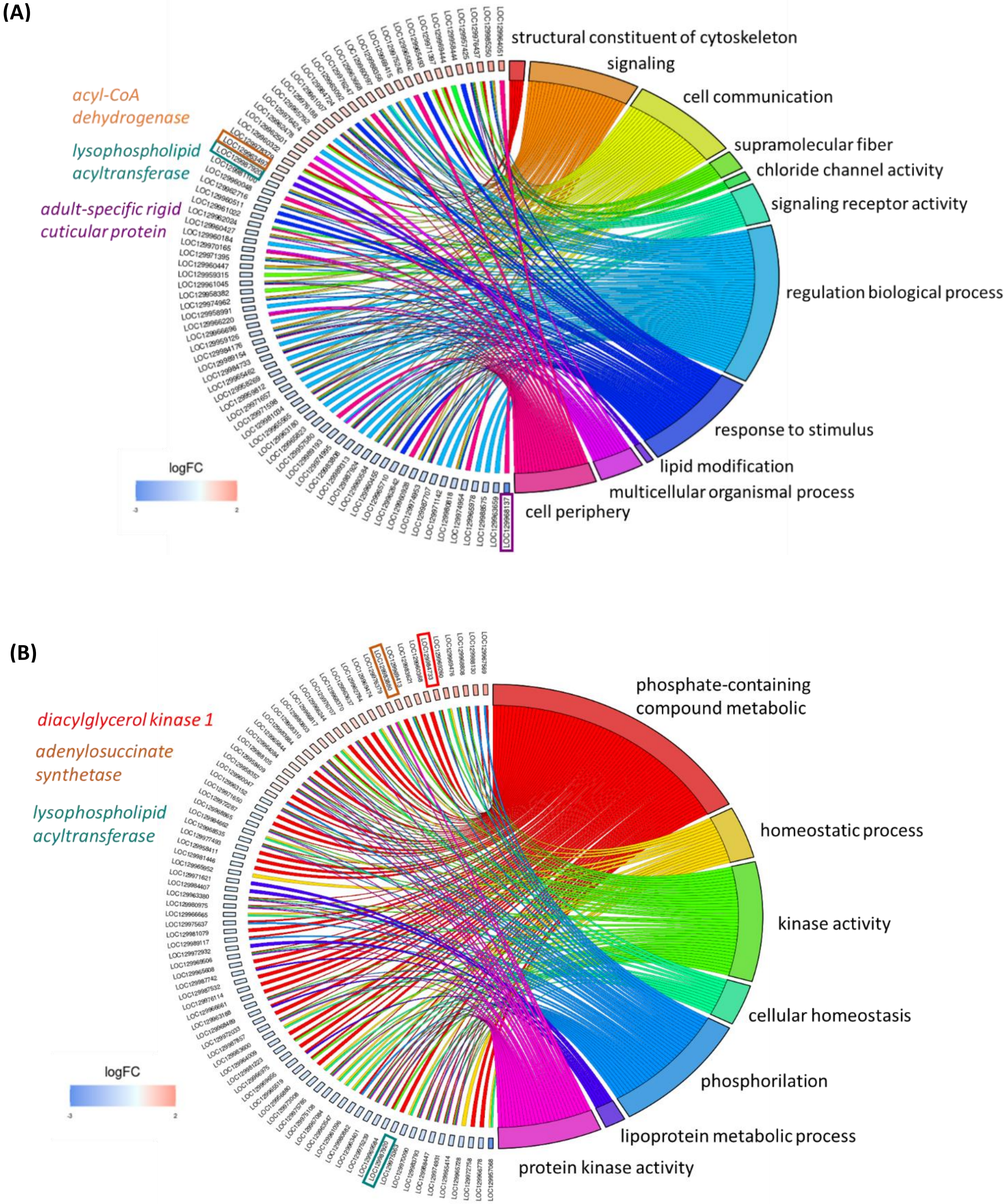

(A)
structural constituent of cytoskeleton
signaling
cell communication
supramolecular fiber
chloride channel activity
signaling receptor activity
regulation biological process
response to stimulus
lipid modification
multicellular organismal process
cell periphery
acyl-CoA dehydrogenase
lysophospholipid acyltransferase
adult-specific rigid cuticular protein
logFC
-3
2
(B)
phosphate-containing compound metabolic
homeostatic process
kinase activity
cellular homeostasis
phosphorilation
lipoprotein metabolic process
protein kinase activity
diacylglycerol kinase 1
adenylosuccinate synthetase
lysophospholipid acyltransferase
logFC
-3
2

(C)

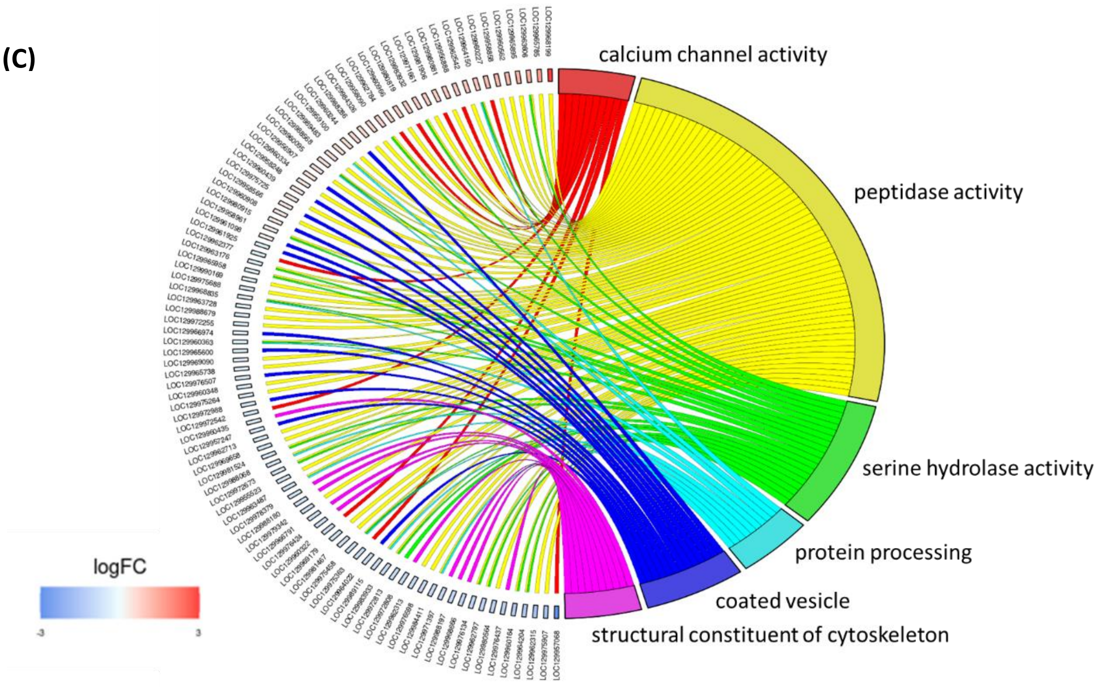

Figure S4. Gene ontology (GO) terms and associated differentially expressed genes DEGs (padj < 0.05) for the **(A)** cold vs moderate, **(B)** warm vs moderate, and **(C)** warm vs cold comparisons. The chord plot shows genes (represented by rectangles with associated LOC ID numbers) that belong to selected GO terms (a full list of GO terms can be found in Table S4) using colored ribbons. Genes are colored and ordered based on their log fold-change value, higher positive log fold-change (red hue) means greater expression in the regime. Select DEGs of interest are highlighted based on log fold-change, connectivity with multiple ontologies, and biological relevance.

Table S5. Kyoto Encyclopedia of Genes and Genomes (KEGG) pathways (FDR of 0.1) for differentially expressed genes between regimes in *Argiope bruennichi* (abru) spiderlings. # refers to the number of genes enriched in the pathway. In bold are the significantly enriched pathways (FDR < 0.05).

Pathway ID	Description	Category	#
abru00270	Cysteine and methionine metabolism	Metabolism	11
abru00130	**Ubiquinone terpenoid-quinone biosynthesis**	Metabolism	5
abru00230	**Purine metabolism**	Metabolism	4
abru00260	Glycine, serine and threonine metabolism	Metabolism	8
abru00410	beta-Alanine metabolism	Metabolism	9
abru00330	Arginine and proline metabolism	Metabolism	10
abru00052	**Galactose metabolism**	Metabolism	11
abru00240	**Pyrimidine metabolism**	Metabolism	12

abru00500	**Starch and sucrose metabolism**	Metabolism	12
abru00380	**Tryptophan metabolism**	Metabolism	10
abru01240	Biosynthesis of cofactors	Metabolism	26
abru00591	Linoleic acid metabolism	Metabolism	8
abru00561	Glycerolipid metabolism	Metabolism	14
abru01200	Carbon metabolism	Metabolism	22
abru00480	Glutathione metabolism	Metabolism	15
abru00564	Glycerophospholipid metabolism	Metabolism	19
abru00590	Arachidonic acid metabolism	Metabolism	13
abru00760	**Nicotinate and nicotinamide metabolism**	Metabolism	7
abru00190	Oxidative phosphorylation	Metabolism	17
abru00983	**Drug metabolism - other enzymes**	Metabolism	15
abru00982	Drug metabolism - cytochrome P450	Metabolism	27
abru04142	Lysosome	Cellular Processes	35
abru04814	**Motor proteins**	Cellular Processes	33
abru04144	Endocytosis	Cellular Processes	34
abru04145	**Phagosome**	Cellular Processes	24
abru04137	**Mitophagy – animal**	Cellular Processes	22
abru04146	Peroxisome	Cellular Processes	18
abru04820	**Cytoskeleton in muscle cells**	Cellular Processes	16
abru04512	**ECM-receptor interaction**	Signaling	12
abru03030	**DNA replication**	Processing	14
abru03018	RNA degradation	Processing	17
abru03430	**Mismatch repair**	Processing	7
abru04130	**SNARE interactions in vesicular transport**	Processing	10
abru03013	Nucleocytoplasmic transport	Processing	20
abru04141	Protein processing in endoplasmic reticulum	Processing	38
abru03040	Spliceosome	Processing	32
abru04120	Ubiquitin mediated proteolysis	Processing	27
abru04068	FoxO signaling pathway	Processing	21
abru04150	mTOR signaling pathway	Processing	24
